# Non‐Thermal Plasma Activation of Gold‐Based Catalysts for Low‐Temperature Water–Gas Shift Catalysis

**DOI:** 10.1002/anie.201612370

**Published:** 2017-04-12

**Authors:** Cristina E. Stere, James A. Anderson, Sarayute Chansai, Juan Jose Delgado, Alexandre Goguet, Willam G. Graham, C. Hardacre, S. F. Rebecca Taylor, Xin Tu, Ziyun Wang, Hui Yang

**Affiliations:** ^1^ School of Chemistry and Chemical Engineering Queens University Belfast David Keir Building Belfast BT9 5AG UK; ^2^ School of Chemical Engineering and Analytical Science The University of Manchester The Mill Manchester M13 9PL UK; ^3^ Surface Chemistry and Catalysis Group School of Engineering University of Aberdeen Aberdeen AB24 3UE UK; ^4^ *y*Departamento de Ciencia de los Materiales e Ingeniería Metalúrgica*y*Química Inorgánica Facultad de Ciencia Universidad de Cádiz 11510 Puerto Real (Cádiz) Spain; ^5^ School of Mathematics and Physics Queens University Belfast Belfast BT7 1NN UK; ^6^ Department of Electrical Engineering and Electronics University of Liverpool Liverpool L69 3GJ UK

**Keywords:** carbon monoxide, heterogeneous catalysis, plasma catalysis, thermodynamic equilibrium, water–gas shift reaction

## Abstract

Non‐thermal plasma activation has been used to enable low‐temperature water‐gas shift over a Au/CeZrO_4_ catalyst. The activity obtained was comparable with that attained by heating the catalyst to 180 °C providing an opportunity for the hydrogen production to be obtained under conditions where the thermodynamic limitations are minimal. Using in situ diffuse reflectance infrared Fourier transform spectroscopy (DRIFTS), structural changes associated with the gold nanoparticles in the catalyst have been observed which are not found under thermal activation indicating a weakening of the Au−CO bond and a change in the mechanism of deactivation.

Many reversible exothermic reactions are of great industrial importance. For instance, the water–gas shift (WGS) reaction [Eq. (1)] is an important process in the production of clean hydrogen for processes such as oil refining, ammonia synthesis or hydrogen fuel cells. The main role of this reaction step is to remove the CO formed during the upstream hydrocarbon reforming reactions while increasing the hydrogen yield. This step is essential since CO is often a poison for downstream processing catalysts [Eq. (1)].

(1) $$\mathrm{CO}\left(g\right)+H{}_{2}O\left(g\right)⇌\mathrm{CO}{}_{2}\left(g\right)+H{}_{2}\left(g\right)$$

Such reversible exothermic reactions come with difficulties in terms of process development since thermodynamics and kinetics have antagonist temperature requirements. From a thermodynamic point of view, only low temperature operation allows access to high equilibrium conversion whilst from a kinetics point a view, the higher the reaction temperature the better. In the specific case of the WGS reaction (Δ*H*°_298_=−41.09 kJ mol^−1^) the thermodynamic equilibrium at high temperature limits the CO conversion, to the point that WGS processes usually require multiple reactors in series with decreasing operation temperatures. In some cases, this is not sufficient to achieve low enough output CO concentrations and a further CO purification step is added. If lower temperature operations could be achieved, significant process intensification and operational cost saving would ensue. Figure 1 illustrates the issue associated with the relationship between catalyst activity and thermodynamic limitations. As the reaction temperature is raised to increase the activity, the equilibrium limitation is approached and limits the achievable conversion.

**Figure 1:**
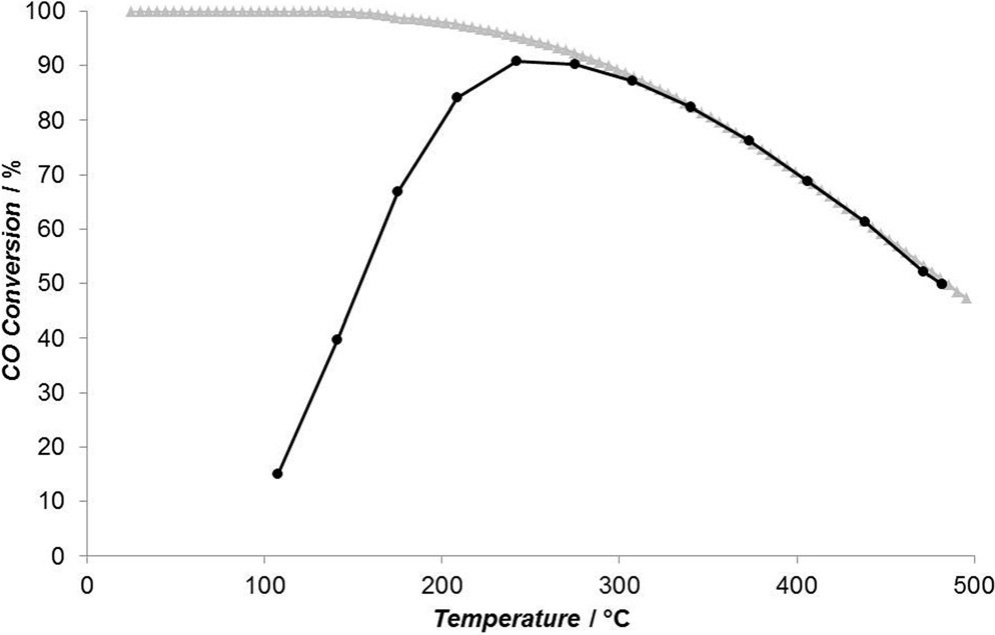
CO conversion as a function of reaction temperature in the full WGS reaction mixture (2.0 % CO, 7.5 % H_2_O, 2.5 % CO_2_, 8.1 % H_2_) with the Au/CeZrO_4_ catalyst (black) and the thermodynamic equilibrium (gray).

Alternatives to thermally activated processes have recently been explored to enhance low temperature operation, amongst which the non‐thermal plasma activation of catalysts^1^, ^2^, ^3^, ^4^ which, in many cases, has been shown to provide high conversions for irreversible exothermic reactions.^1^, ^2^, ^3^, ^4^, ^5^, ^6^, ^7^, ^8^, ^9^, ^10^, ^11^, ^12^ Examples include decomposition of volatile organic compounds (VOCs) and hydrogenation reactions.^4^, ^5^, ^6^, ^7^, ^8^, ^9^, ^12^, ^13^ Non‐thermal plasmas have also been widely used with endothermic processes such as reforming processes^14^, ^15^, ^16^, ^17^ leading to high H_2_ and CO selectivities.^14^, ^15^, ^16^ Sekine et al. showed an enhancement in activity with increasing current on applying an electric field to the forward water‐gas shift reaction when the catalyst was also heated to 423–873 K.^2^ The water–gas shift reaction has also been reported in close proximity of a plasma source, that is, post plasma catalysis, to enhance the hydrogen production following a plasma activated ethanol reforming system.^17^ Therein, the water–gas shift reaction used the heat generated from the plasma system to transform the reformed products.

Close coupling of plasmas with heterogeneous catalysts presents many advantages, including the possibility of opening up alternative reaction pathways from the plasma‐generated species^13^ and structural changes of the active phase due to the plasma; however, the activation mechanism depends on many factors including the type of catalyst, reactants, reaction conditions.^3^, ^4^, ^18^, ^19^, ^20^, ^21^ The present study demonstrates that a reversible exothermic reaction of significant industrial importance can be performed at close to room temperature using heterogeneous catalysis and dielectric‐barrier discharge (DBD) plasma activation; and thus overcoming the equilibrium limitations whilst maintaining high catalytic activity.

Light‐off experiments with a full WGS reaction mixture (2.0 % CO, 7.5 % H_2_O, 2.0 % CO_2_, 8.1 % H_2_) over a Au/CeZrO_4_ catalyst showed high WGS activity between 100 and 240 °C (Figure 1); with the CO conversion reaching a maximum of 91 % at 240 °C. Similar results were reported for a 2.0 wt % Au/CeZrO_4_ by Pilasombat et al.^22^ and Tibiletti et al.^23^ Importantly, CO conversion was observed at low temperatures, for example, 15 % conversion at 100 °C. Figure 2 reports a comparison of the CO conversions obtained with an empty reactor, the pure CeZrO_4_ support and the Au/CeZrO_4_ catalyst under plasma conditions (7.5 kV and 22.5 kHz) using the full WGS feed. The empty reactor and the CeZrO_4_ support both gave very low conversions about 7 %. A significant increase in CO conversion was observed when the gold catalyst was used, reaching 70 % under these conditions. Whilst no heat source was applied, the application of the plasma led to an increase in the reactor temperature. This rise was not related to the exothermicity of the reaction since, in all three cases, the measured temperature of the system was approximately 115 °C, irrespective of the extent of conversion obtained. The enhancement effect of the plasma‐activated catalytic system over the thermal system is clear. At 115 °C, only 20 % CO conversion was found over the gold catalyst under thermal activation (Figure 1) compared with 70 % conversion under plasma conditions. In order to explore the importance of the Joule heating during plasma activation, a simplified mixture, containing only CO and H_2_O was also tested. Under these conditions the CO conversion obtained under plasma activation was close to 90 %. To achieve a similar performance thermally requires temperatures above 400 °C, as shown in Figure S1 in the Supporting Information. Therefore, the effect of plasma on the CO conversion is not solely associated with a Joule heating effect.

**Figure 2:**
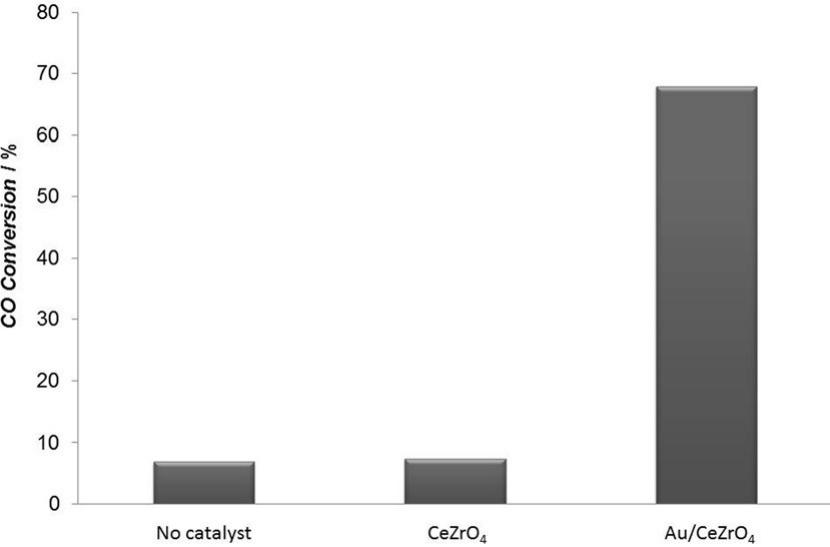
CO conversion under plasma conditions (7.5 kV, 22.5 kHz) in the full WGS reaction mixture (2.0 % CO, 7.5 % H_2_O, 2.5 % CO_2_, 8.1 % H_2_) over the CeZrO_4_ support, the Au/CeZrO_4_ catalyst and in the absence of a catalyst.

The variation of CO conversion with applied voltage is shown in Figure S2. A significant increase in the conversion was observed from <40 % at 6.0 kV to 70 % at 7.5 kV. The calculated specific energy input for an applied voltage of 7 kV was 9.2 W. No change in the selectivity was observed for the different voltages tested or under thermal conditions. In all cases, the reaction was selective to CO_2_, with mass balances of 97 %±3 % (Table S1) which is consistent with the previous studies under thermal control.^22^, ^23^ Furthermore, temperature‐programmed oxidation of the used samples confirmed that little carbon deposition took place and that the carbon‐containing species evolution could be attributed to carbonate decomposition after both thermal and plasma reactions^24^ (Figure S3). This dependence on applied voltage is in contrast with Sekine et al. which may reflect the application of a DC compared with AC electric field.^2^

Despite the excellent activity at relatively low reaction temperatures, Au‐based catalysts are often affected by significant deactivation with time on stream.^24^, ^25^, ^26^, ^27^, ^28^, ^29^ Herein, a similar deactivation behaviour was observed for similar starting conversions of CO under thermal (178 °C) and plasma conditions (Figure S4). This deactivation was previously attributed ^[27]^ to surface hydrolysis leading to a loss of the gold–support interaction and the rate of deactivation was observed to increase with increasing water concentration. A similar behavior was observed under plasma conditions (Figure S5). Interestingly, whilst the trends in the initial rate of deactivation are similar under both thermal and plasma activation, the effect on gold differs.^30^ Longer‐term deactivation experiments have also been performed over 35 h time on stream (Figure S6). The initial rapid deactivation rate was over 2.5 % h^−1^ (Figure S5); however after 10 h reaction the rate slowed to 0.6 % h^−1^ and, thereafter, the rate remained constant.

Following the reaction under plasma activation, transmission electron microscopy showed an increase in the average gold particle size from 0.7 to 1.8 nm (Figure S7), suggesting sintering of gold may be a cause of deactivation under these conditions. This is in contrast with previously reported data for the thermally activated reaction which showed no significant change in particle size.^26^, ^27^ Furthermore, a decrease of the surface area from 73 m^2^ g^−1^ (fresh catalyst) to 56 m^2^ g^−1^ after plasma exposure has also been observed. However, it is important to note that the activity of the gold catalyst was still significant even after 500 min reaction time with 57 % CO conversion observed for the Au/CeZrO_4_ catalyst under plasma conditions under full WGS reaction conditions. Characterization of the catalyst before and after plasma exposure did not show changes in the XRD patterns (Figure S8). Only CeO_2_ and ZrO_2_ features were present^31^ with no Au peaks suggesting that gold particles were well dispersed on the surface of support and the size of gold particles was smaller than the detection limit of XRD, smaller than 5 nm^25^, ^31^ which is in agreement with the TEM results.

Figures S9 and S10 show the X‐ray photoelectron spectral (XPS) lines of Au 4f, and Ce 3d regions for the Au/CeZrO_4_ catalyst and a summary of the XPS binding energy data is given in Table S2. Binding energies of 84.0 eV and 86.3 eV were observed for the Au 4f_7/2_ corresponding to Au^0^ and Au^3+^; however, no changes were observed before and after the plasma reaction. The only significant change observed was a decrease in the Ce^3+^/(Ce^3+^+Ce^4+^) surface ratio from 0.34 to 0.17 which indicates some surface oxidation of the support had occurred when the catalyst was exposed to plasma.

In order to further probe the gold active site under the plasma activation, in situ diffuse reflectance infrared Fourier transform spectroscopy (DRIFTS) was used. This used a previously reported DRIFTS reactor setup where the plasma was set to only penetrate a small portion of the sample, therefore, ensuring differential conditions for the spectroscopic measurements.^32^ The inlet gas composition was set to the simplified mixture forward WGS feed (2 % CO, 7.5 % H_2_O), therefore, ensuring that the CO_2_ observed was entirely derived from the plasma activation. The in situ DRIFTS spectra were recorded as a function of time during three plasma on‐off cycles over the Au/CeZrO_4_ catalyst (see Figure S11). The formation and disappearance of gas phase CO_2_ (2300 and 2400 cm^−1^) associated with a decrease in the IR bands of both CO and H_2_O when plasma was switched on confirmed the activity of the catalyst for the WGS reaction under these conditions. A comparison of the spectrum recorded at 150 °C under thermal conditions, that is, no plasma activation, and the spectrum obtained when the plasma was on showed the presence of formates in the region 3000–2500 cm^−1^, carbonates and formates in the region 1700 and 800 cm^−1^ and carbonyl bands in the region 2200–2000 cm^−1^.^27^, ^33^, ^34^ Moreover, a significant reduction in the carbonate bands observed under plasma conditions may indicate that the CO_2_ release was promoted. The presence of surface carbonates blocking the redox sites on the catalyst have been proposed as potential causes of deactivation.^28^, ^29^, ^35^, ^36^, ^37^ Importantly, the presence of the plasma reduces the carbonate features present which indicates that this is probably not the cause of the deactivation in the present system.

In order to characterize the state of the catalyst further, an examination of the carbonyl bands has been undertaken. These features have been used extensively to characterize the state of the surface gold sites.^23^, ^38^, ^39^ For example, IR bands at 2110–2090 cm^−1^ have been assigned to CO on metallic Au particles (Au^0^−CO)^25^, ^38^, ^39^, ^40^ whereas bands between 2120 and 2180 cm^−1^ are thought to be due to CO adsorbed on Au^δ+^. In addition, bands in the region of 2050–1950 cm^−1^ have been assigned to CO adsorbed on very small gold cluster/negatively charged Au.^25^ Figure S12 compares the carbonyl region (2200–2000 cm^−1^) under thermal conditions and also during plasma on‐off cycles. For each spectrum, the gas phase CO was subtracted from the original spectra recorded during the reaction. During thermal activation at 150 °C, the predominant feature was the band at 2095 cm^−1^ associated with the CO adsorbed on Au^0^, specifically at the step sites or the perimeter of Au nanoparticles, consistent with previous studies.^23^, ^27^, ^34^ A small contribution of Au^δ+^−CO species at 2120 cm^−1^ was also observed. Exposure of the fresh catalyst to WGS feed under plasma activation (first cycle) led to the appearance of similar adsorbed features, with Au^0^−CO species more dominant than the Au^δ+^−CO species (Figure 3). Previous studies showed that under thermal conditions, metallic Au is the most stable and an active species for the water‐gas shift reaction.^23^, ^26^, ^27^, ^41^, ^42^ No CO bands were observed when the pure support was examined under similar conditions. On extinguishing the plasma over the catalyst, a significant decrease in the adsorbed CO was observed predominantly associated with the band at 2095 cm^−1^, that is, associated with adsorption on the gold nanoparticles. A small decrease was also observed in the band at 2120 cm^−1^ attributed to adsorption on partially oxidized gold.

**Figure 3:**
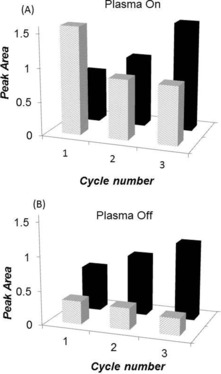
Changes in the peak area of the Au^0^ (2095 cm^−1^, gray) and Au^δ+^ (2120 cm^−1^, black) species during A) plasma on and B) plasma off cycles.

On consecutive cycling of the plasma, an overall decrease in the Au^0^−CO band was observed, whereas the intensity of the band at 2120 cm^−1^ increased with cycling, both during application of the plasma and in its absence. This change in the nature of the adsorption of the CO is consistent with the change in the electron microscopy before and after the plasma treatment. Therein, particle growth of the gold was observed which would decrease the amount of interfacial gold atoms responsible for the band at 2095 cm^−1^. Previous studies have shown that the size of this feature under thermal conditions can be related to the activity of the catalyst.^26^, ^27^, ^43^ This may also be the case under plasma conditions which shows deactivation with time on stream; the fact that after three cycles this band has decreased considerably, yet the catalyst is still active would suggest that this species is, indeed, being converted into a less active form (Au^δ+^). Under the plasma conditions, it is postulated that reactive oxygen species (ROS)^1^, ^44^, ^45^, ^46^ formed through the activation of the water leads to oxidation of the gold. This reactive oxygen species, which may be OH, for example, generated in the gas phase plasma will also increase the hydrolysis of the gold‐support interface and lead to the loss of the interfacial gold species, represented by the band at 2095 cm^−1^. Previously reported density functional theory (DFT) calculations have demonstrated that the decoration of the surface with hydroxy groups significantly weakens the metal–support interaction and a dewetting of the gold nanoparticle. This loss of the interfacial gold which is exacerbated by the sintering of the gold particles, leads to a deactivation of the catalyst. As neither sintering or oxidation of the surface is observed under thermal conditions this highlights the significantly higher reactivity of the water derived species which is activated by ionization in the gas phase rather than by the oxygen vacancies on the support. These changes are also consistent with the XPS results which show surface oxidation of the support on exposure to the plasma, that is, a reduction in the surface concentration of oxygen vacancies. The formation of oxygen vacancies are catalyzed by surface metal sites and, therefore, the loss of surface gold would also reduce their surface concentration.

To obtain a better understanding of the mechanisms governing the high catalytic activity achieved under plasma conditions, DFT was used to calculate the free energy profile of the WGS over Au(100) and Au(111) for both redox and carboxyl pathways shown in Figures S15 and S16, respectively. The results strongly suggest that the water activation is the rate‐determining step on gold surfaces, which is in agreement with previous results.^47^ Water has been observed to be activated in the gas phase under plasma conditions to form, for instance, OH and H_2_O^+^ species.^1^, ^44^, ^45^, ^46^, ^48^ The former is thought to be a key intermediate from the water dissociation on the surface of gold and the subsequent reaction to form COOH before releasing CO_2_. Prior formation of the OH before adsorption would reduce the effective barriers, using the method reported by Wang et al.,^49^ from 2.87 to 0.73 eV on Au(111) and from 2.44 to 0.99 eV on Au(100). Furthermore, the activation barriers for the dissociation of H_2_O^*x*^ as a function of the charge *x* over gold was calculated and was found to be negative for cationic water (Figure S17), suggesting that H_2_O^+^ is not stable and will dissociate into OH and H spontaneously. Therefore, the plasma activated low temperature WGS is likely to be due to the facile activation of H_2_O reducing the overall activation barrier. It should be noted although the role of the support has not been taken into account in these calculations, activation of water is also thought to be a key step in the WGS process at the interface between the gold and the support. Therefore, the activation barriers are likely to be even lower in the presence of the oxide under plasma activation.

In conclusion, the reported results clearly demonstrate that it is possible to decouple the thermodynamics of the WGS process from the kinetics using a dielectric‐barrier discharge activated heterogeneous catalyst system that enables the reaction to occur at low temperature. In situ diffuse reflectance infrared spectroscopy coupled with plasma activation lead to the formation of various types of both gas phase and surface species including CO (ads), CO_2_ (g and ads), formates, carbonates, and water. Similar species were reported to be formed under various WGS reaction conditions over a variety of catalysts. The plasma could affect the rate‐determining step on the gold surface by activating the water in the gas phase. Most importantly, the DRIFTS study demonstrated an impact of the DBD on the structural properties of the gold leading to a significant change in the adsorption properties of CO indicative of a weakening of the Au−CO bond.

## Conflict of interest

The authors declare no conflict of interest.

## Supporting information

As a service to our authors and readers, this journal provides supporting information supplied by the authors. Such materials are peer reviewed and may be re‐organized for online delivery, but are not copy‐edited or typeset. Technical support issues arising from supporting information (other than missing files) should be addressed to the authors.

Supplementary
