# Supplementary material for: Non‐Thermal Plasma Activation of Gold‐Based Catalysts for Low‐Temperature Water–Gas Shift Catalysis
Source: Angew Chem Int Ed Engl. 2017 Apr 12;56(20):5579–83. doi: 10.1002/anie.201612370 (PMC5485072; doi:10.1002/anie.201612370)
Supplement: Supplementary file 1 — Supplementary [file ANIE-56-5579-s001.pdf]

Angewandte Chemie
Eine Zeitschrift der Gesellschaft Deutscher Chemiker

Supporting Information

Non-Thermal Plasma Activation of Gold-Based Catalysts for Low-Temperature Water–Gas Shift Catalysis

Cristina E. Stere, James A. Anderson, Sarayute Chansai, Juan Jose Delgado, Alexandre Goguet, Willam G. Graham, C. Hardacre, S. F. Rebecca Taylor, Xin Tu, Ziyun Wang, and Hui Yang*

anie_201612370_sm_miscellaneous_information.pdf

Experimental

The catalytic activity, stability and selectivity tests with a gold-based catalyst have been carried out in a fixed bed flow reactor using both conventional heating and non-thermal plasma (NTP) conditions. Spectroscopic insights on the catalyst behaviour during and after exposure to plasma were performed in an *in situ* Diffuse Reflectance Infrared Fourier Transform (DRIFT) cell previously developed [C.E. Stere et al. *ACS Catal.* **2015**, *5*, 956].

Catalyst Preparation

All chemicals were obtained from Sigma-Aldrich and used as received. A 1.2% Au/$Ce_{0.5}Zr_{0.5}O_4$ catalyst, designated as Au/$CeZrO_4$, was prepared by deposition-precipitation method (DP) described in [R. Pilasombat et al. *Catal. Today*, **2012**, *180*, 131]. The ceria-zirconia ($Ce_{0.5}Zr_{0.5}O_4$) support was synthesized by sol-gel method from $Ce(NO_3)_3{\cdot}6H_2O$ and $ZrO(NO_3)_2{\cdot}xH_2O$. Firstly, the appropriate amounts of each nitrate precursor: 7.4 g $Ce(NO_3)_3{\cdot}6H_2O$ and 3.9 g $ZrO(NO_3)_2{\cdot}xH_2O$ were mixed and dissolved in 150 cm^3 of deionized (DI) water. An aqueous NH_4OH was added under constant stirring (400 rpm) until the pH reached a value of 9.0. After filtering and washing, the resulting gel was dried at 100 ^oC for 12 h and then calcined at 500 ^oC for 4 h.

The synthesized ceria-zirconia ($CeZrO_4$) powder was slurried in de-ionised water under constant stirring (500 rpm) and warmed up to 60 ^oC. The pH was adjusted to 8.0 by adding 0.05 M aqueous solution of sodium carbonate (Na_2CO_3). A 1.2 mM $HAuCl_4{\cdot}xH_2O$ (SIGMA-ALDRICH) stock aqueous solution was prepared, and then added slowly into the support slurry at ~1 cm^3 min^{-1} under constant stirring (500 rpm), while adding 0.05 M aqueous solution of Na_2CO_3 to maintain the pH around 8.0. After the gold precursor addition was finished, the slurry was stirred 400 rpm at 60 ^oC for 1 h. The resulting solid was filtered and washed with 175 cm^3 deionized water per gram of precipitate to remove the residual chloride and then dried at 70 ^oC for 6 h.

Catalyst characterization

The specific surface area and pore volume of the fresh and used Au/$CeZrO_4$ catalyst were measured by BET using a Micromeritics ASAP 2020 and were found to be 73 $m^2 g^{-1}$ and 0.103 $m^3 g^{-1}$, respectively for the fresh catalyst and for the used catalyst 56 $m^2 g^{-1}$ and 0.096 $m^3 g^{-1}$, respectively.

The gold loading was measured by ICP-OES and found to be 1.2 wt%.

Structural and spectroscopic measurements were carried out both on the fresh samples and after exposure to plasma. Transmission Electron Microscopy (TEM) and high resolution transmission electron microscopy (HRTEM) were performed on a JEOL2010F instrument with electron acceleration energy of 200 kV. Powder X-ray diffraction (XRD) studies were carried out using a PANalytical X'Pert Pro X-ray diffractometer at room temperature. The X-ray source used was copper Kα with the wavelength of 1.5405 Å. The diffractograms were recorder from 15° to 90 ° with a step size of 0.017° and time per step is ca. 10.2 s. The scan speed used for the ex-situ analysis was 0.21° s^{-1}. XPS analysis was carried out using Kratos AXIS Ultra DLD apparatus equipped with monochromated Al K_α X-ray source, a charge neutralizer and a hemispherical electron energy analyzer with a pass energy of 160 eV. Background subtraction was performed using a Shirley background and CasaXPS software was employed to treat the data. The XPS was referenced to the C 1s feature at 285.0 eV.

Catalytic activity with conventional heating or plasma

The catalyst (250 - 425μm particle size) was held in place between quartz wool plugs in a 6mm OD quartz tube with a K-type thermocouple placed in the centre of the catalyst bed to monitor the reaction temperature during light-off experiments. The gases in the feed were controlled individually using mass flow controllers (Area, Advanced Energy), while water vapour was produced by means of a CEM (Bronkhorst E7000) system. Condensation was prevented by heat tracing the gas lines before and after the reactor. The gas exiting the reactor was analysed using a gas

chromatograph (Perkin Elmer Clarus 5000) equipped with a stainless steel Haysep DB 100/120 mesh packed column (30 ft, OD=1/8", ID=2.0mm), a thermal conductivity detector (TCD) and a flame ionisation detector (FID) fitted with a methanizer to allow measurement of CO and CO_2.

The plug-flow reactor was adapted for plasma-generation by replacing the internal thermocouple with a 0.5mm O.D. tungsten wire that acted as a ground electrode and a stainless steel mesh on the outer of the reactor (anode). In order to prevent arcing due to increase thermal conductivity of the dielectric medium at high humidity, insulation of the ground electrode was achieved by enclosing the tungsten wire into a 0.7 mm sealed quartz capillary. The plasma was ignited by using an alternating current power source (PVM500 model) and the electrical parameters were monitored using an oscilloscope (Tektronix TBS1062) that was connected to the reactor through a high voltage probe (Tektronix, P6015). The applied voltage was 7.5 kV (unless otherwise stated) at a frequency of 22.5 kHz. The catalyst was carefully packed in the dielectric barrier discharge region to ensure a direct contact with the plasma-generated species and to take advantage of any possible local heating effects. The reaction temperature was monitored by an IR probe (ProUSB, Calex, PU301). A photograph of the reactor under plasma operation is reported in figure S2 (A).

The forward WGS simplified feed gas stream consisted of 2.0% CO, 7.5% H_2O (unless otherwise stated) and the full mixture, aimed to incorporate both the forward and reverse WGS components, consisted of 2.0% CO, 2.5% CO_2, 7.5% H_2O and 8.1% H_2. In all cases, 0.5% Kr was used as internal standard and argon as balance gas to keep a constant gas space velocity over the catalyst bed (50 mg) of 60,000 h^{-1}.

Thermally activated light off tests were carried out by increasing the reaction temperature at 1 °C min^{-1} from 100 °C to 450 °C, with 1 h dwell time at 450 °C.

The time on stream stability of the catalyst was tested over 500 min at constant temperature or non-thermal plasma (NTP) conditions (7.5 kV, 22.5 kHz), respectively. The temperature (178 °C) was chosen to match the initial CO conversion under NTP conditions and to provide a direct comparison of the two systems.

Equations 1 and 2 were used to calculate the CO conversion and the CO_2 selectivity,

respectively. Each point is an average of three consecutive measurements, with a calculated error of ± 2.60% for CO conversion and ± 2.00% for CO_2 selectivity.

$$\%CO\ conversion = \frac{[CO]_{inlet} - [CO]_{outlet}}{[CO]_{inlet}} \times 100 \quad \text{(Equation 1)}$$

$$\%CO_2\ Selectivity = \frac{CO_2\ Production}{[CO]_{inlet} - [CO]_{outlet}} \times 100 \quad \text{(Equation 2)}$$

Equilibrium conversion values were based on thermodynamic limitations and obtained from the Convergence tool of Aspen Plus 8.0.

NTP-DRIFTS experiments

The experimental setup for the *in situ* IR experiments was described in detail elsewhere. [C.E. Stere et al. *ACS Catal.* **2015**, 5, 956] The catalyst was loaded into the IR cell and pre-treated at 150 °C for 30 min under Ar to ensure any residual water was removed from the catalyst before being exposed to the plasma. The temperature was then decreased to room temperature and the NTP was generated in close contact with the surface of the catalyst. The simplified forward WGS feed was allowed to flow through the T-shape quartz tube to the plasma region and exit the cell through the catalyst bed. A picture of the NTP-DRIFTS setup is reported in Figure S11 (B). The power electrode was driven at a peak voltage of 6.0 kV and the pulse frequency was kept at 22.0 kHz to avoid arcing between the external electrode and the collector.

IR spectra were recorded every 60 s with a resolution of 4 cm^{-1} and were analyzed with the OPUS software. The IR data are reported as log 1/*R* (“absorbance”), with *R* = I/I_0, where *R* is the sample reflectance, I the intensity measured under reaction conditions, and I_0 the intensity measured on the sample under a flow of argon, immediately prior to the introduction of the reactant mixture. For the analysis of the Au-CO bands, all spectra were corrected for the residual gas phase CO and the peaks fitted with a number of Gaussian peak shapes (Figure S12).

Density functional theory calculations

All the DFT calculations were carried out with a periodic slab model using the Vienna ab initio simulation program (VASP) [a) G. Kresse, J. Furthmüller, *Phys. Rev. B* **1996**, *54*, 11169; b) G. Kresse, J. Furthmuller, *Comp. Mater. Sci.* **1996**, *6*, 15; c) G. Kresse, J. Hafner, *Phys. Rev. B* **1994**, *49*, 14251; d) G. Kresse, J. Hafner, *Phys. Rev. B* **1993**, *47*, 558.]. The generalized gradient approximation (GGA) was used with Perdew-Burke-Ernzerhof (PBE) [J. P. Perdew, K. Burke, M. Ernzerhof, *Phys. Rev. Lett.* **1996**, *77*, 3865.] exchange-correlation functional. Projector-augmented wave (PAW) method [a) G. Kresse, D. Joubert, *Phys. Rev. B* **1999**, *59*, 1758; b) P. E. Blöchl, *Phys. Rev. B* **1994**, *50*, 17953.] was utilized to describe the electron-ion interactions and the plane-wave basis expansion cut-off was set to 450 eV. 3×3×1 Monkhorst-Pack *k*-point mesh sampling was used for Brillouin zone integrations. All the adsorption geometries were optimized using a force-based conjugate gradient algorithm, while transition states (TSs) were located with a constrained minimisation technique [a) A. Michaelides, Z. P. Liu, C. J. Zhang, A. Alavi, D. A. King, P. Hu, *J. Am. Chem. Soc.* **2003**, *125*, 3704; b) Z. P. Liu, P. Hu, *J. Am. Chem. Soc.* **2003**, *125*, 1958; c) A. Alavi, P. J. Hu, T. Deutsch, P. L. Silvestrelli, J. Hutter, *Phys. Rev. Lett.* **1998**, *80*, 3650.]. It is widely accepted that the support plays an important role in water gas shift [Z.-J. Zhao, Z. Li, Y. Cui, H. Zhu, W. F. Schneider, W. N. Delgass, F. Ribeiro, J. Greeley, *J. Catal.* **2017**, *345*, 157.]. To decompose the effect of plasma, we only considered the metallic gold surfaces in our DFT calculations. The Au(111) and Au(100) were chosen as the model surfaces for water gas shift. 4-layer and 6-layer models were used for the Au(100) and Au(111), respectively. In the optimization and transition state searching, the 2 lower layers were fixed and 2 upper layers were relaxed for Au(111), while we fix the 3 lower layers of Au(100) and leave the 3 upper layers relaxed. A ~10Å vacuum region was placed on all the models mentioned above. The charges on water molecule were modelled by changing the valence electrons in DFT calculations. It is worth mentioning that under realistic plasma conditions, the electrons may not be at equilibrium state. Thus, our DFT calculations are only used to uncover the effects of H_2O^+ in water gas shift.

Figures and tables

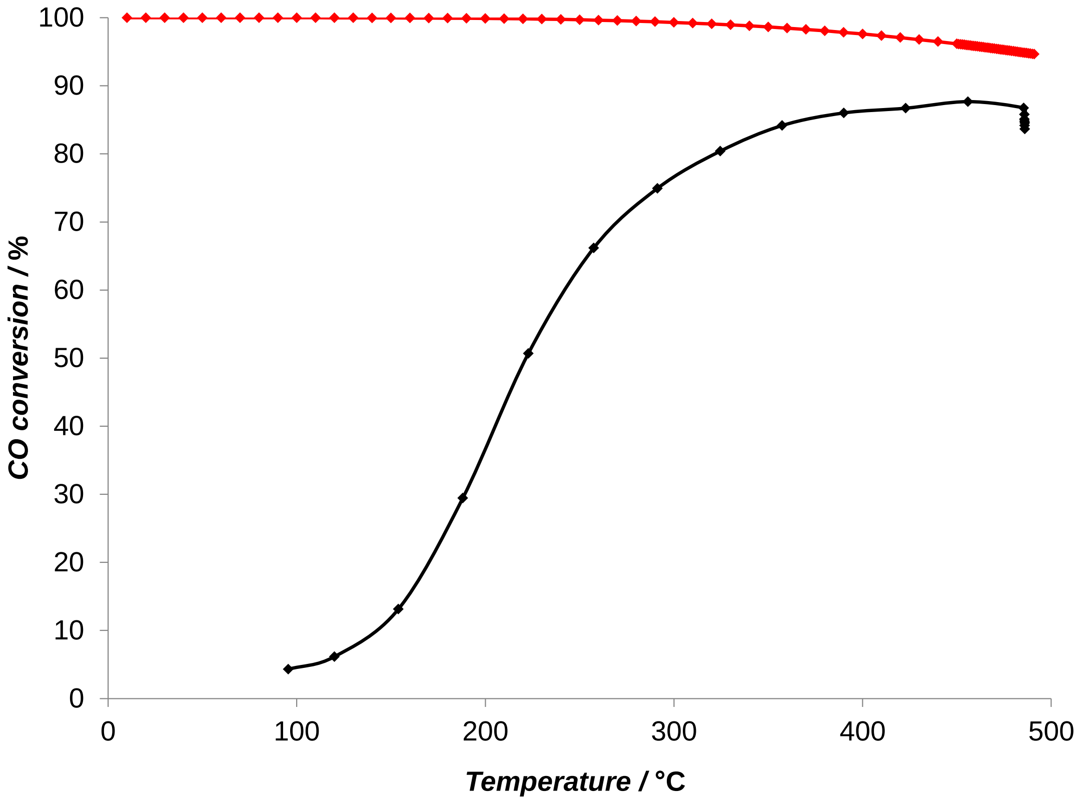

Figure S1. CO conversion as a function of reaction temperature in a simplified mixture (Forward WGS) (2.0% CO, 10% H_2O) over 2% Au/$CeZrO_4$ catalyst (black) and the thermodynamic equilibrium (red).

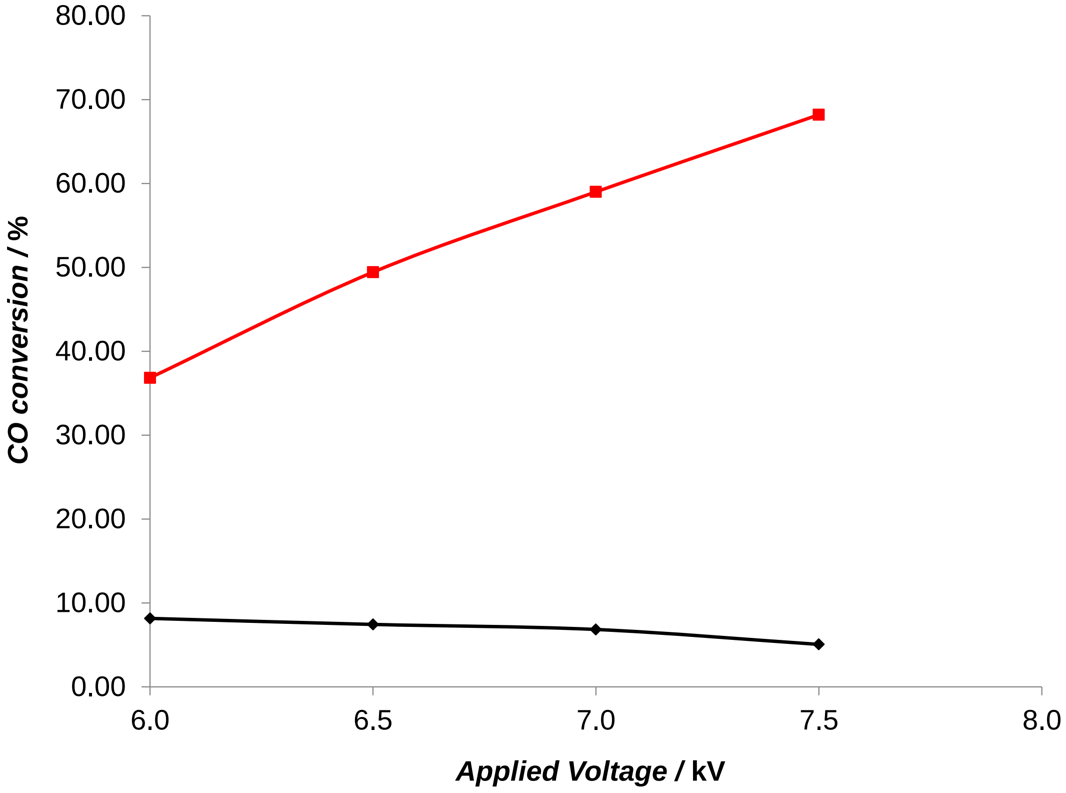

Figure S2: CO conversion as a function of applied voltage in the full WGS reaction mixture

(2.0% CO, 7.5% H_2O, 2.5% CO_2, 8.1% H_2) over 2% Au/$CeZrO_4$ catalyst (red) compared to plasma alone (black).

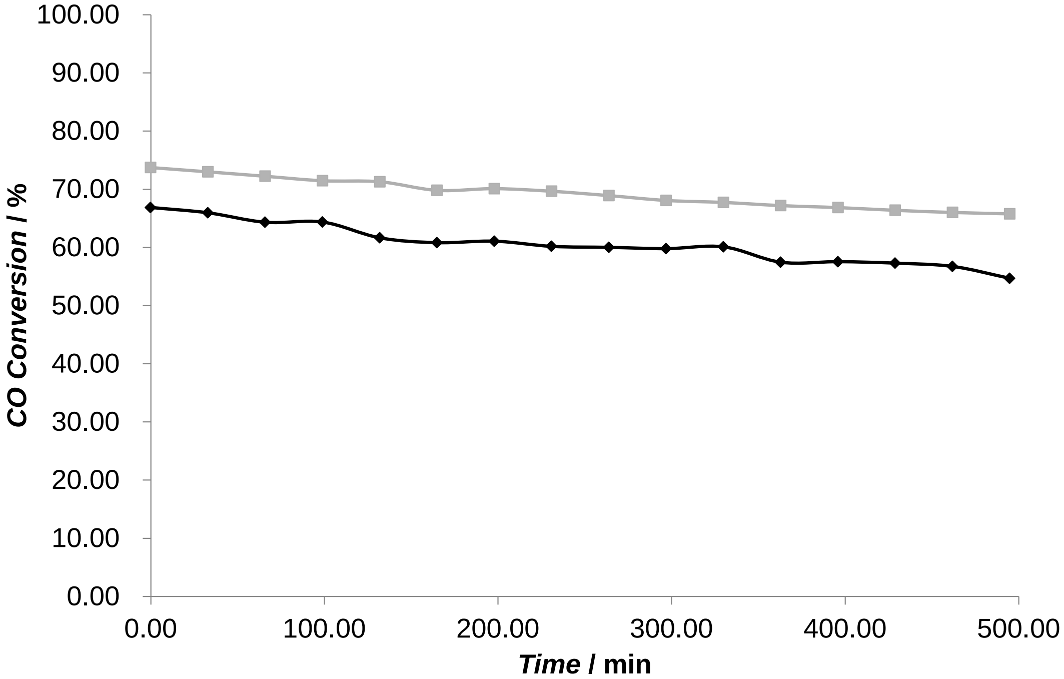

Figure S3: Comparison of CO conversion as a function of reaction time with conventional heating (grey) at 178 ^{o}C and under non-thermal plasma (black) (7.5 kV, 22.5 kHz) in the full WGS reaction mixture (2.0% CO, 7.5% H_2O, 2.5% CO_2, 8.1% H_2) over 2% Au/$CeZrO_4$ catalyst.

Table S1: Selectivity tests at 178 ^{o}C and under NTP conditions (6-7.5 kV, 22.5 kHz) in the full WGS reaction mixture (2.0% CO, 7.5% H_2O, 2.5% CO_2, 8.1% H_2) over 2% Au/$CeZrO_4$ catalyst.

**Experimental condition**	**CO Conversion %**	**CO_2 Production %**	**CO Consumption %**	**CO_2 Selectivity %**
**178 ^{o}C**	73.73	1.43	1.47	97.27
**6 kV**	35.80	0.71	0.72	98.61
**6.5 kV**	49.40	0.97	0.99	97.98
**7 kV**	59.00	1.15	1.18	97.46
**7.5 kV**	68.20	1.31	1.36	96.32

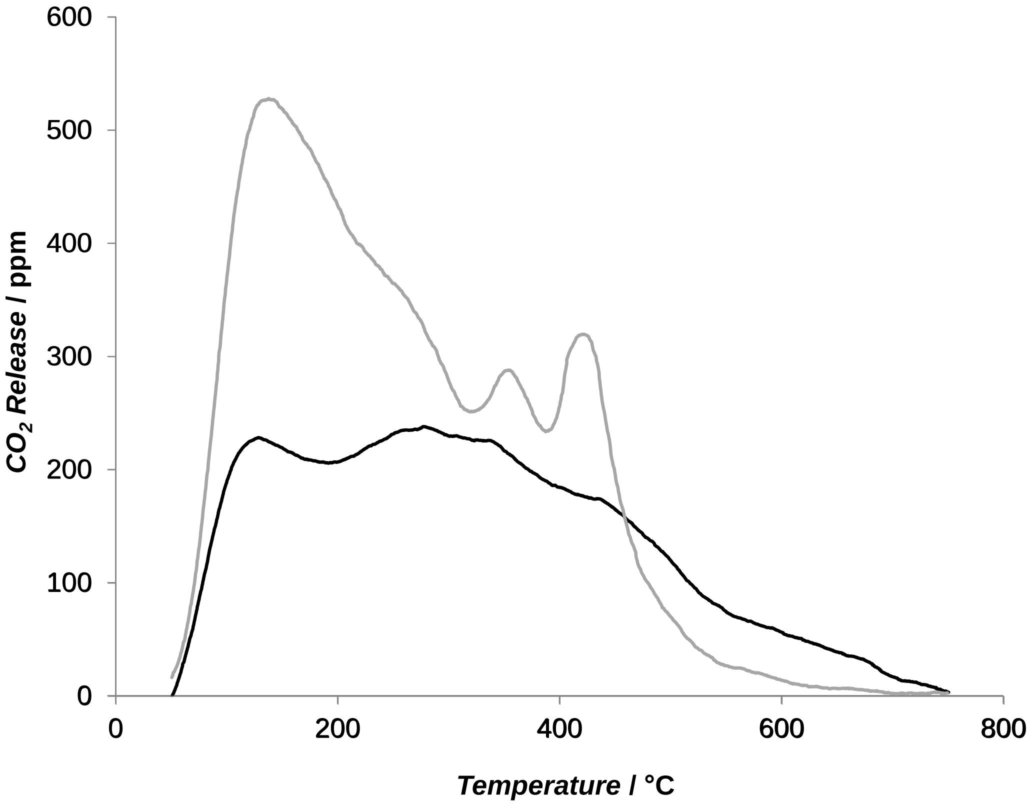

Figure S4: TPO profiles of 2% Au/$CeZrO_4$ catalyst after a WGS light-off test (black) and plasma (grey) test (2.0% CO, 7.5% H_2O, 2.5% CO_2, 8.1% H_2).

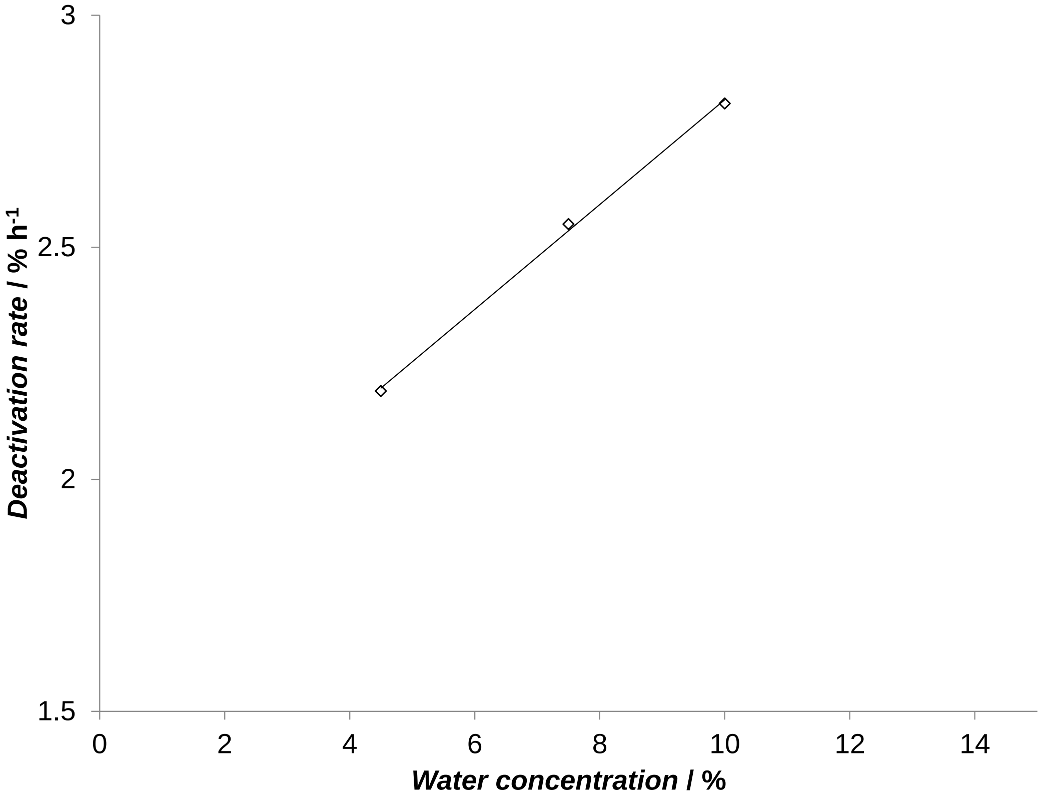

Figure S5: Initial rate of catalyst deactivation as a function of water content of the WGS mix over 2% Au/$CeZrO_4$ catalyst in the presence of plasma.

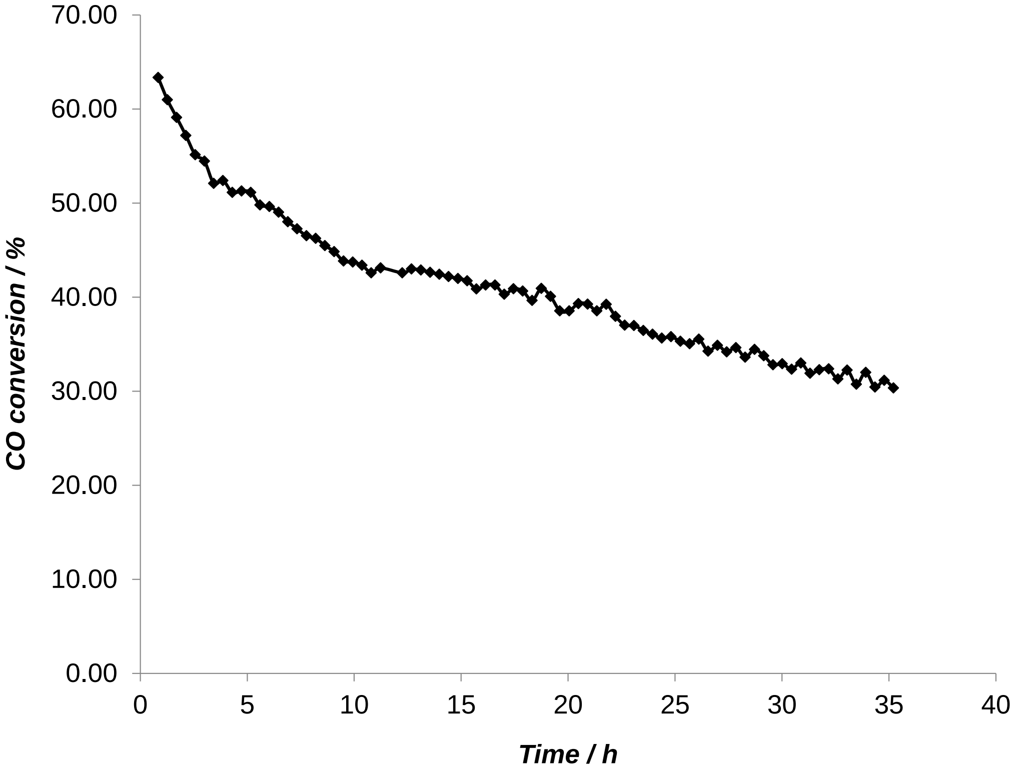

Figure S6: CO conversion as a function of reaction time under non-thermal plasma (black) (7.5 kV, 22.5 kHz) in the full WGS reaction mixture (2.0% CO, 7.5% H_2O, 2.5% CO_2, 8.1% H_2) over 2%Au/$CeZrO_4$ catalyst.

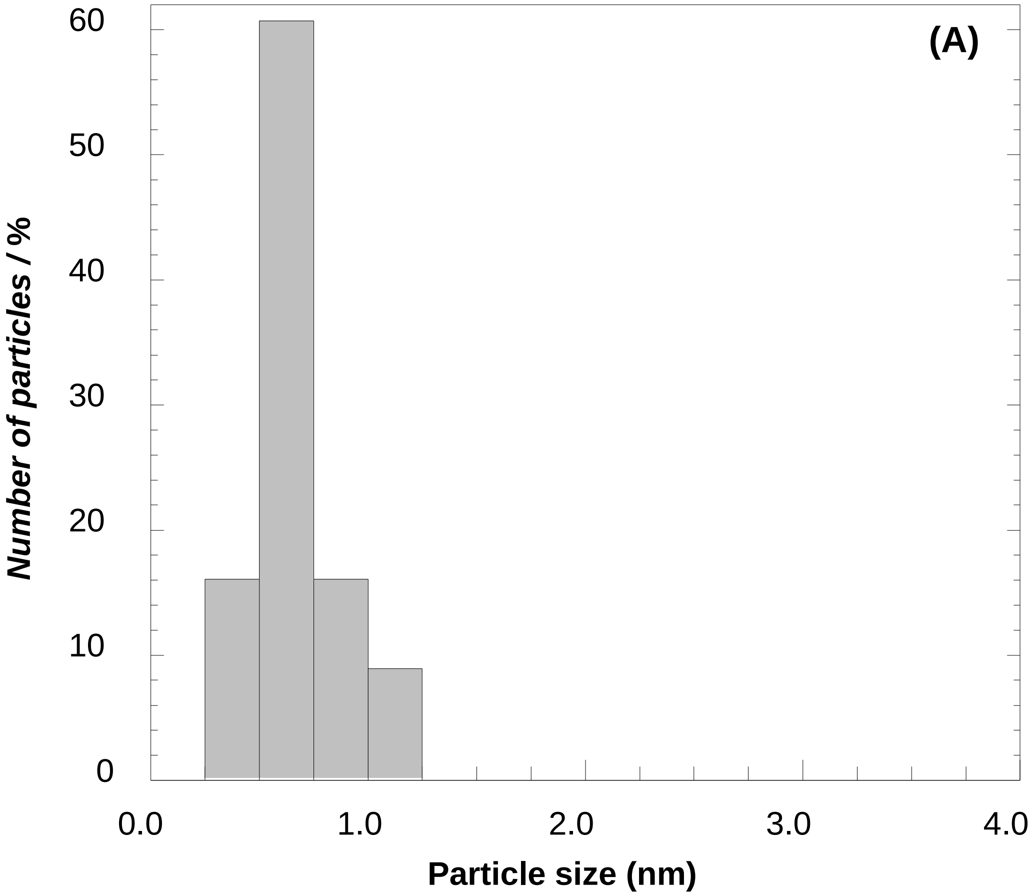

(A)
Number of particles / %
60
50
40
30
20
10
0
0.0
1.0
2.0
3.0
4.0
Particle size (nm)

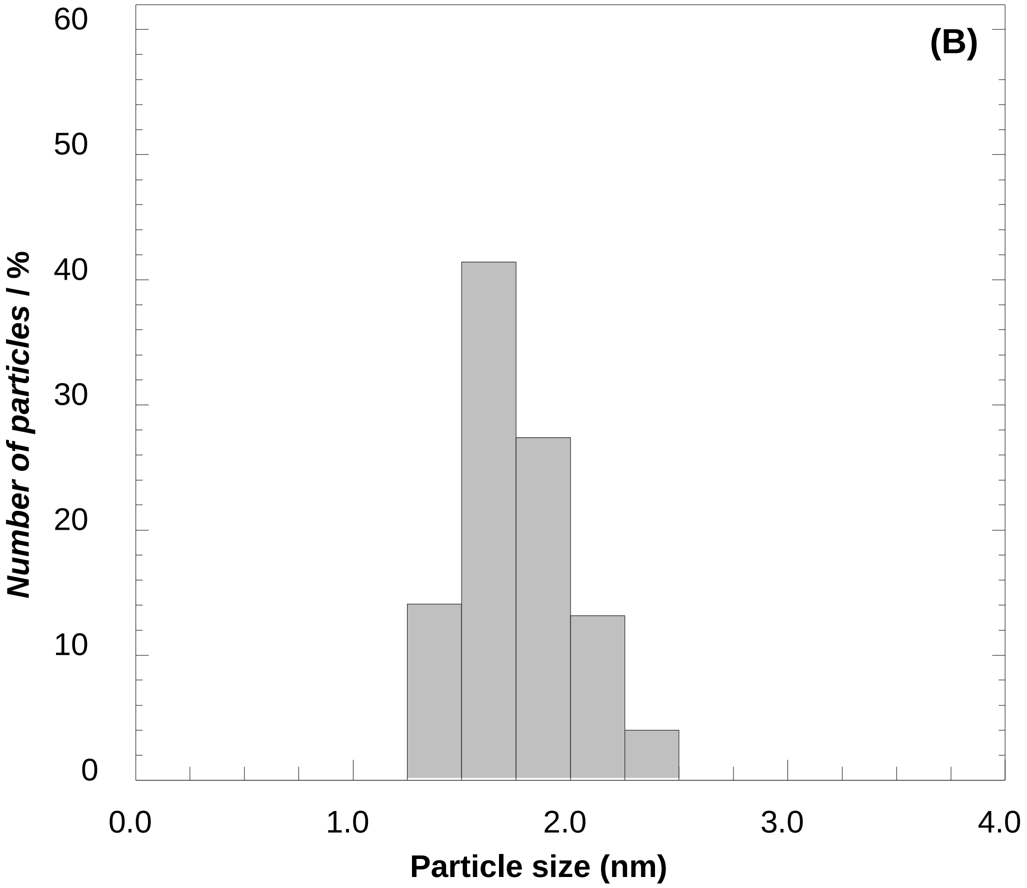
(B)
Number of particles / %
0
10
20
30
40
50
60
0.0
1.0
2.0
3.0
4.0
Particle size (nm)

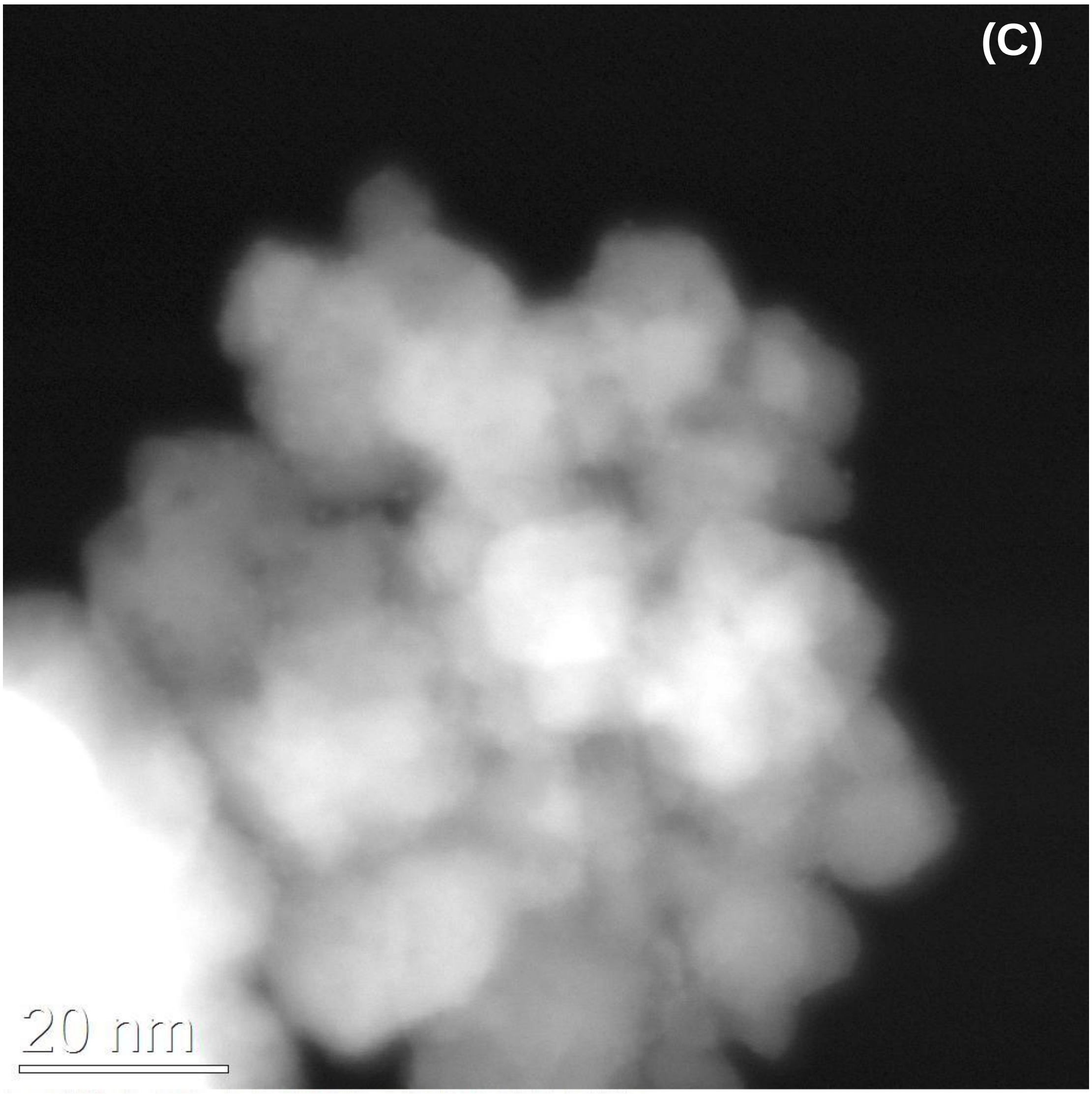
(C)
20 nm

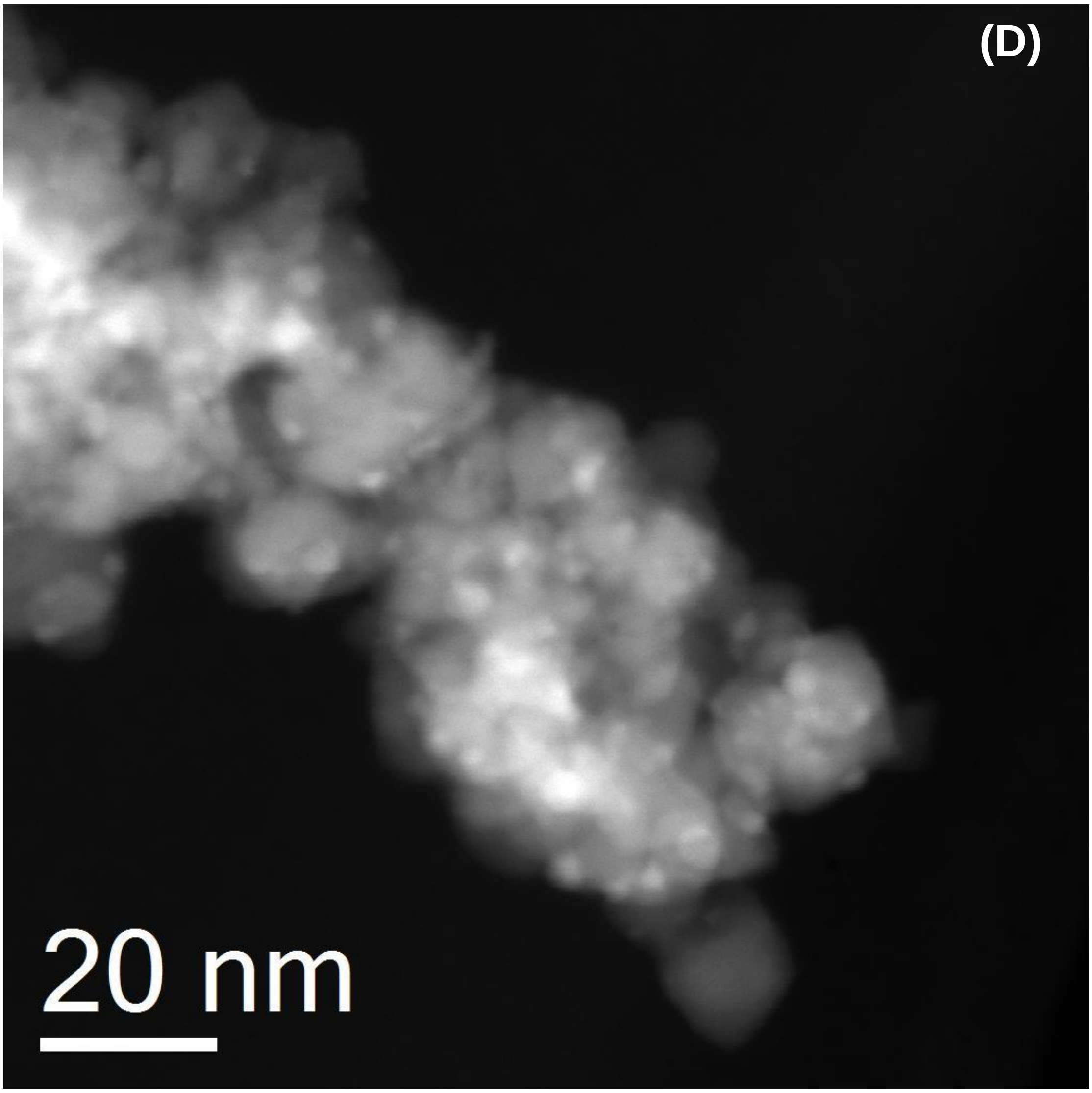

Figure S7: Particle size distribution of the fresh 2% Au/$CeZrO_4$ catalyst (A) and of the 2% Au/$CeZrO_4$ catalyst after NTP test (B); HAADF-TEM of the fresh 2% Au/$CeZrO_4$ catalyst (C) and of the 2% Au/$CeZrO_4$ catalyst after NTP test (D).

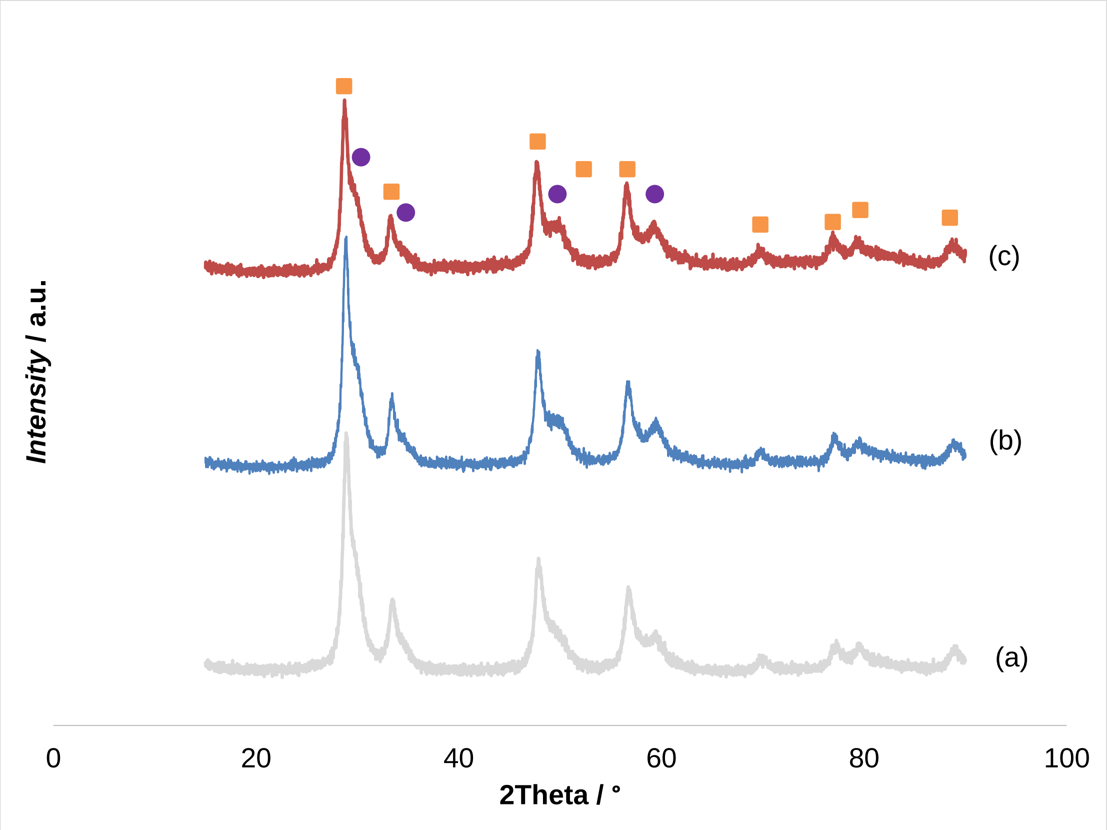

Figure S8: XRD patterns of samples: (a) $CeZrO_4$ support, (b) fresh Au/$CeZrO_4$, (c) Au/$CeZrO_4$ after NTP test. Crystalline phase: CeO_2 (●) and ZrO_2 (■).

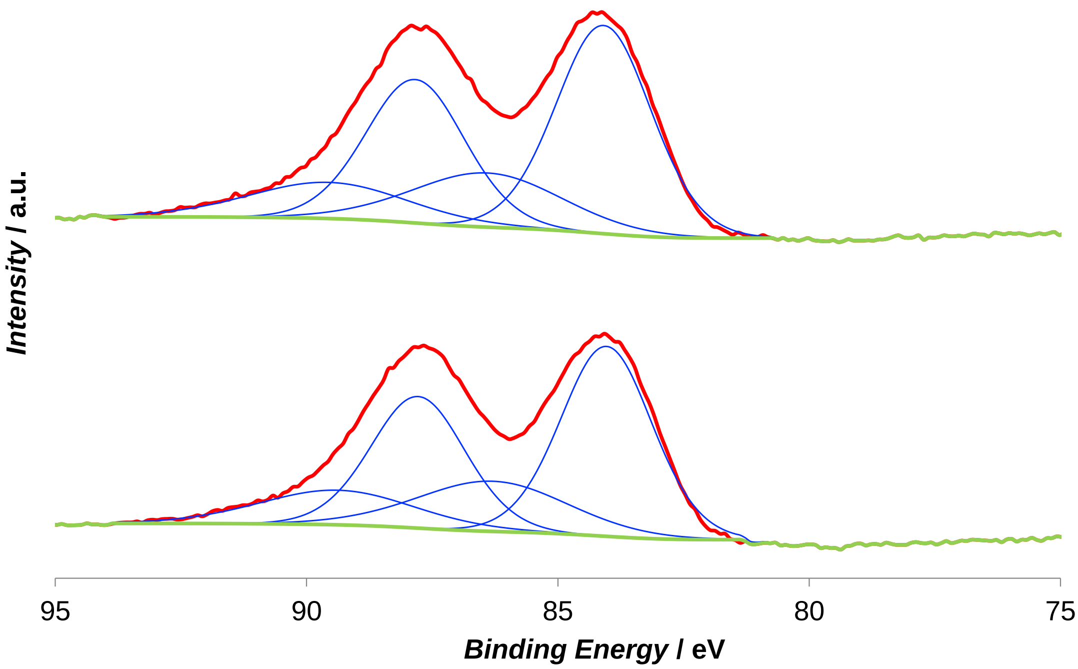

Figure S9: Fitted Au 4f XPS spectrum: (a) fresh 2% Au/$CeZrO_4$ and (b) 2% Au/$CeZrO_4$ after NTP test.

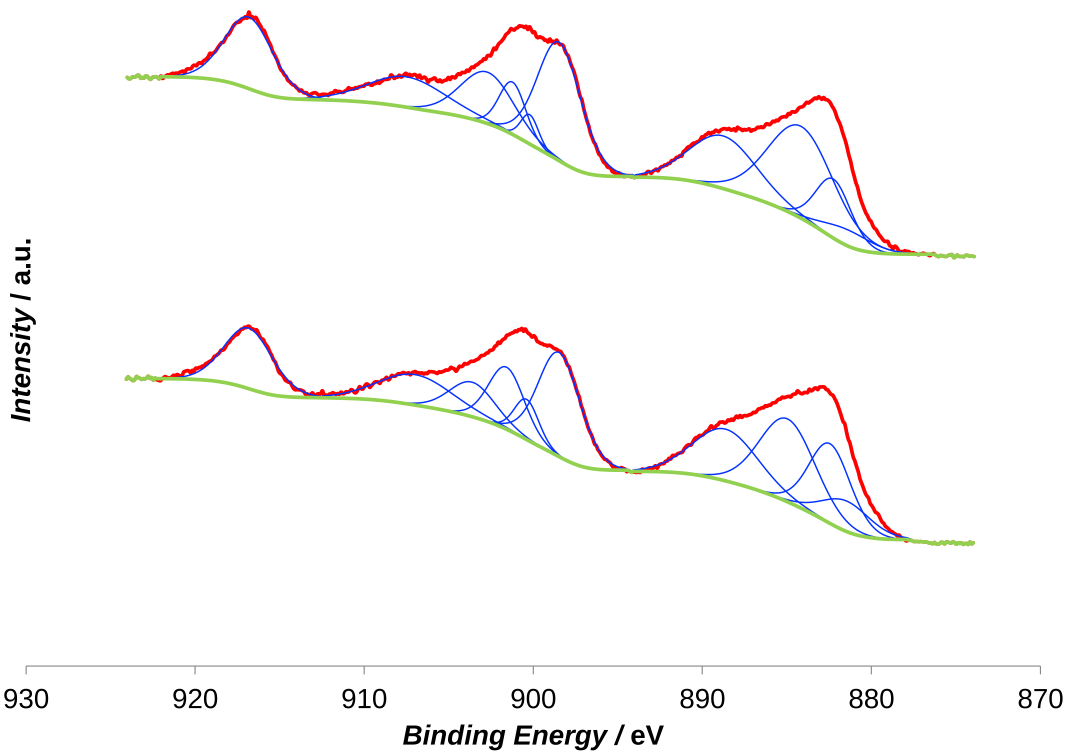

Figure S10: Fitted Ce 3d XPS spectrum: (a) fresh 2% Au/$CeZrO_4$ and (b) 2% Au/$CeZrO_4$ after NTP test.

Table S2: The binding energy of Au and Ce before and after NTP treatment.

Metal	Orbit	Binding Energy (Before NTP Treatment)	Binding Energy (After NTP Treatment)
Au	4f 7/2	84.0 eV<br>86.3 eV	84.1 eV<br>86.4 eV
Ce	3d 5/2	879.7 eV<br>882.2 eV<br>884.9 eV<br>889.1 eV<br>898.4 eV	879.6 eV<br>882.3 eV<br>884.6 eV<br>888.1 eV<br>898.0 eV

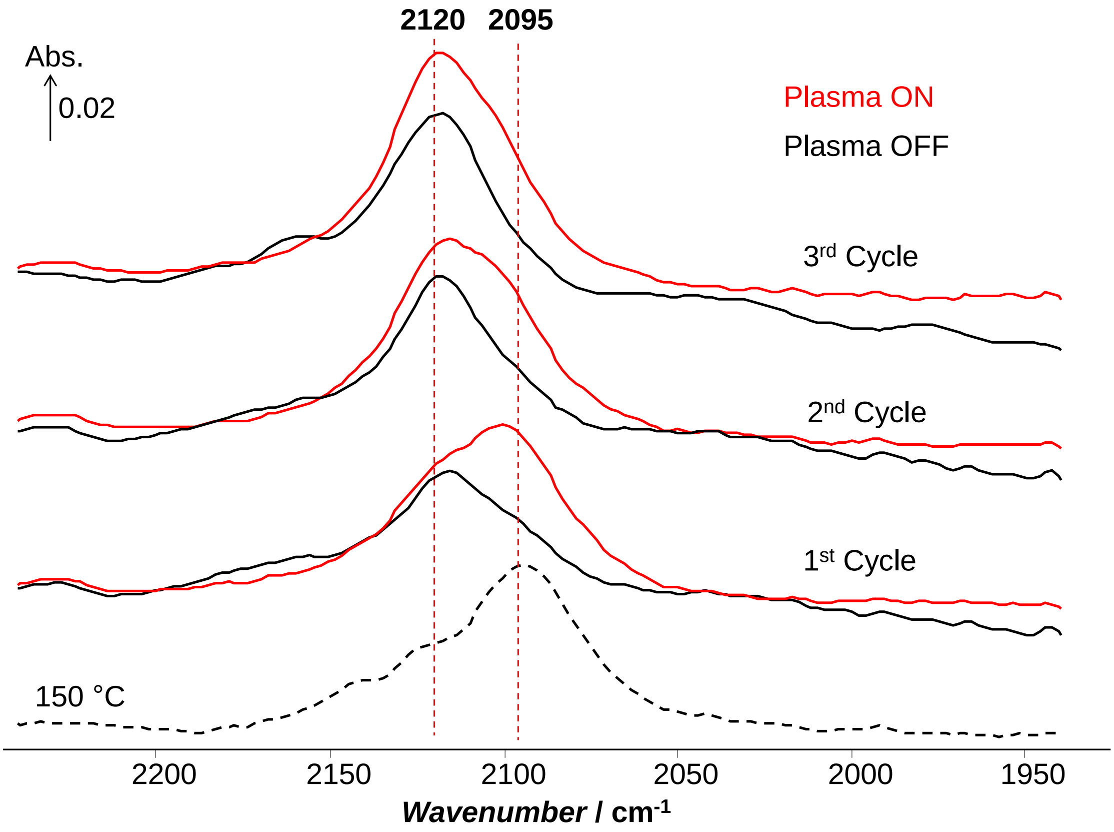

Figure S11: FTIR spectra on the adsorbed and gaseous species recorded during 3 plasma on-off (6 kV, 22.0 kHz) cycles in the simple WGS reaction mixture (2.0% CO, 7.5% H_2O) over 2% Au/$CeZrO_4$ catalyst. Gas phase CO subtracted.

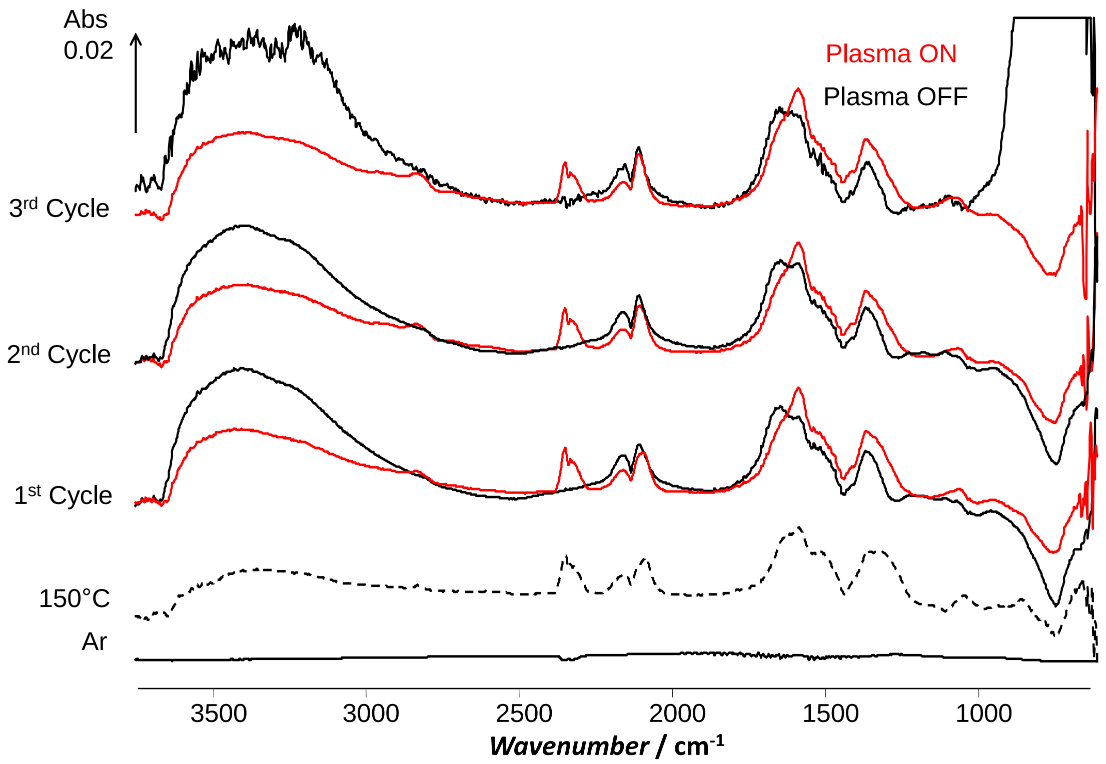

Figure S12: FTIR spectra of the surface and gas phase species recorded during three plasma on-off (6 kV, 22.0 kHz) cycles in the simple WGS reaction mixture (2.0% CO, 7.5% H_2O) over 2% Au/$CeZrO_4$ catalyst.

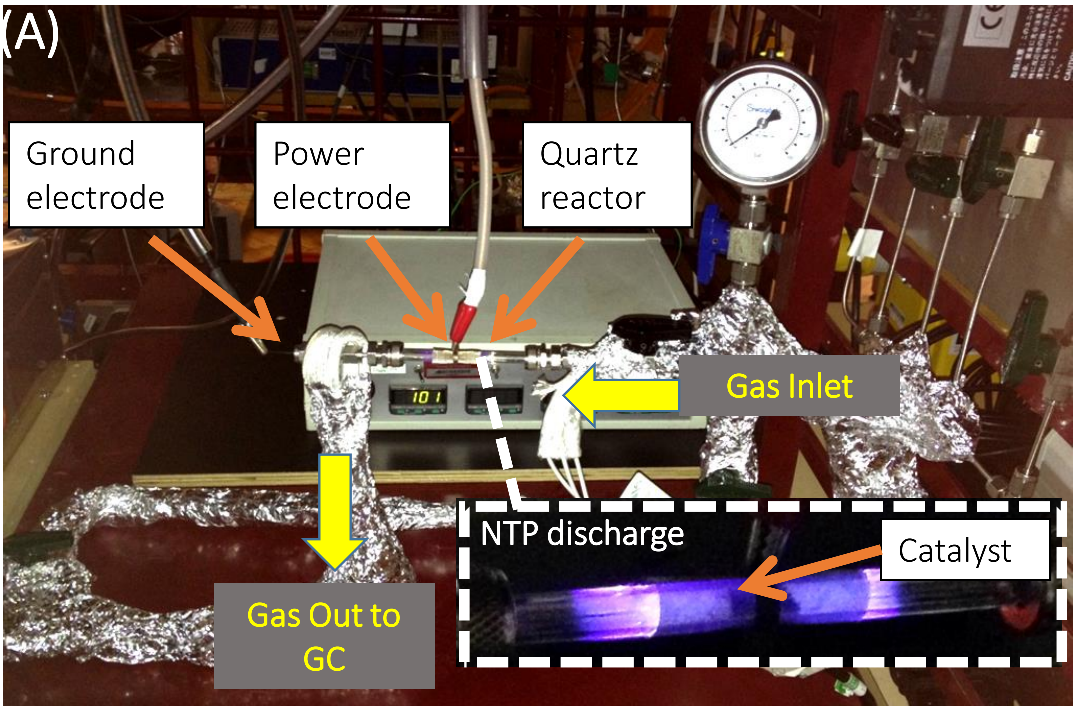

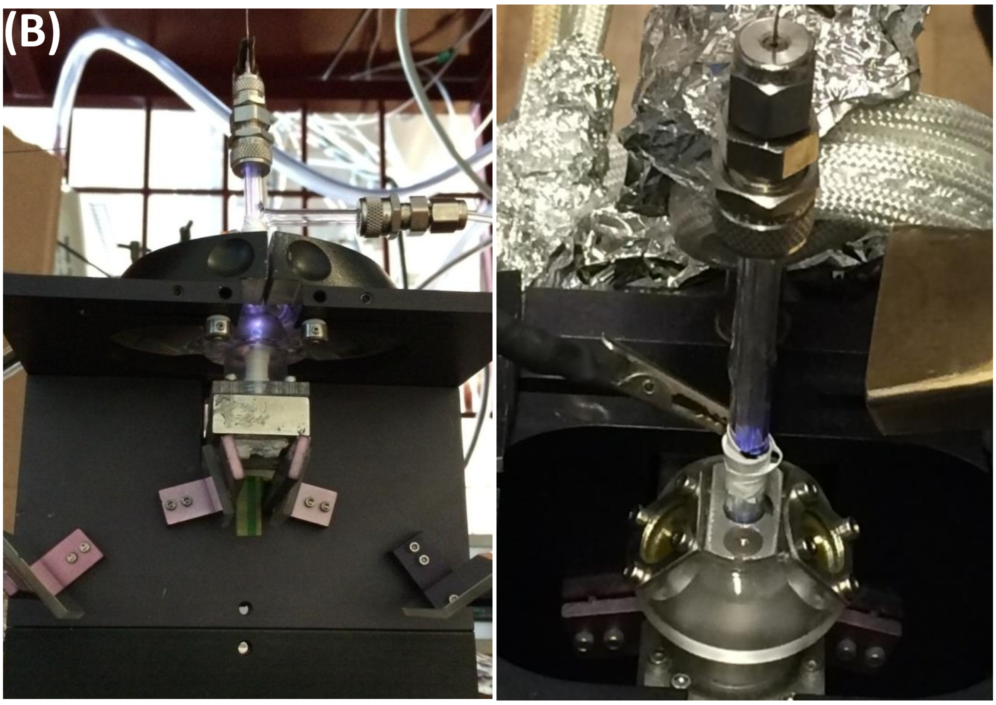

Figure S13: (A) NTP setup for activity tests; (B) NTP-DRIFTS setup.

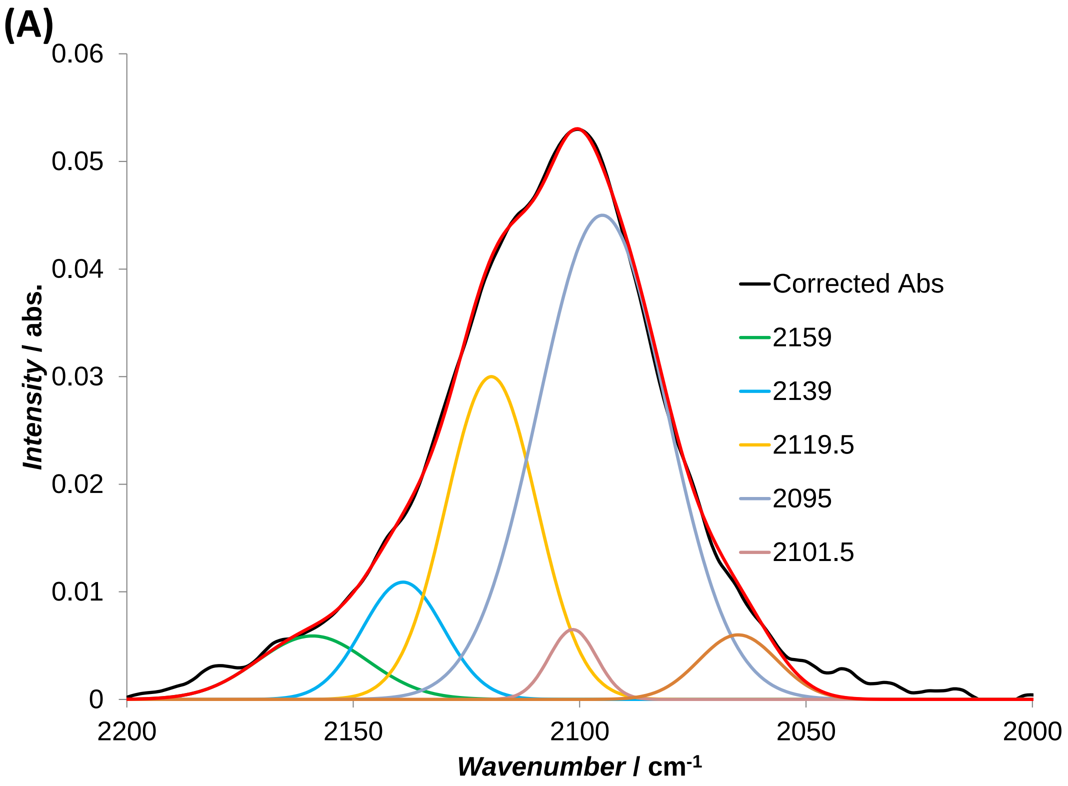

(B)

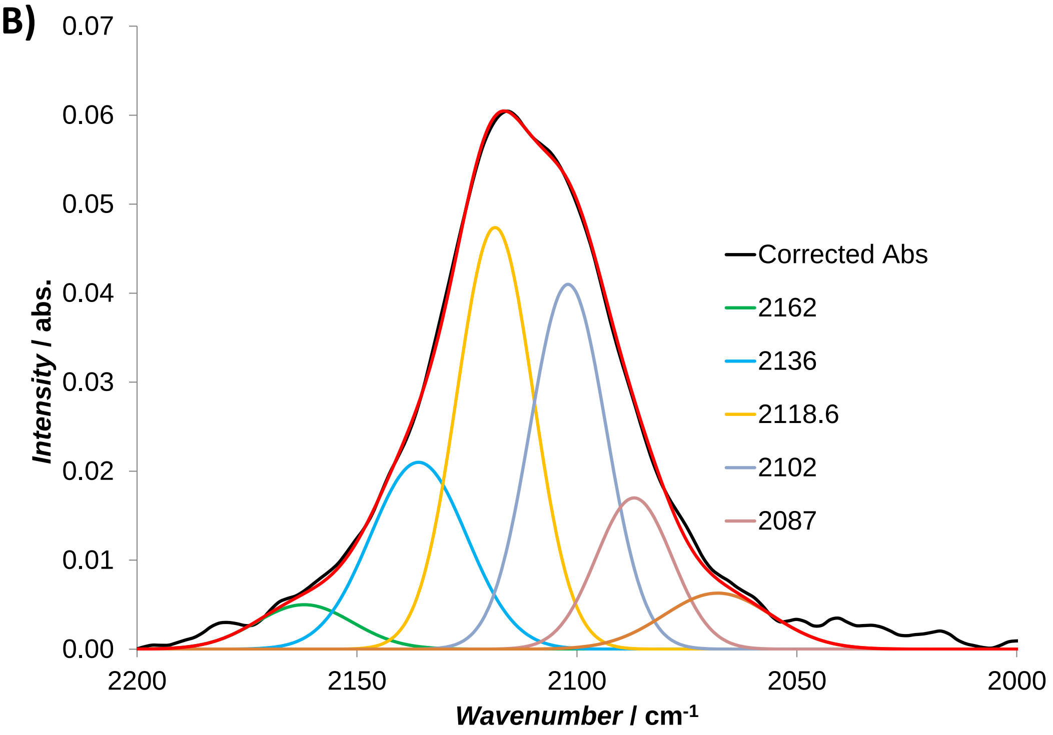

0.07
0.06
0.05
0.04
0.03
0.02
0.01
0.00
Intensity / abs.
2200
2150
2100
2050
2000
Wavenumber / cm-1
Corrected Abs
2162
2136
2118.6
2102
2087

(C)

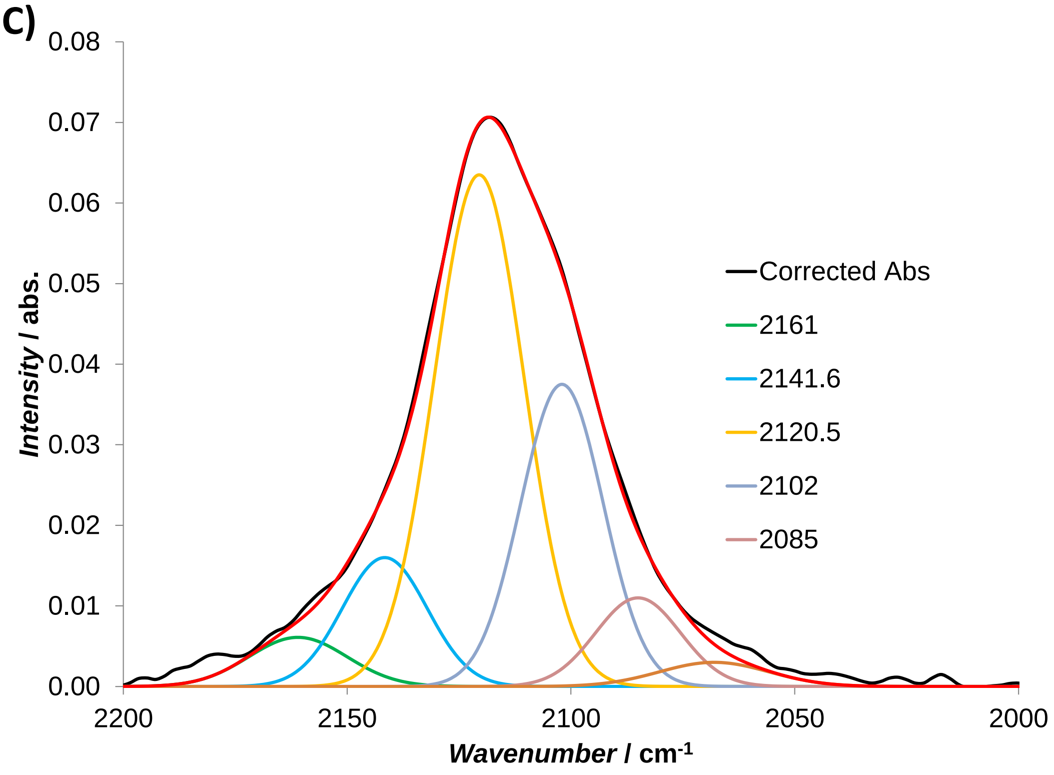

0.08
0.07
0.06
0.05
0.04
0.03
0.02
0.01
0.00
Intensity / abs.
2200
2150
2100
2050
2000
Wavenumber / cm-1
Corrected Abs
2161
2141.6
2120.5
2102
2085

(D)

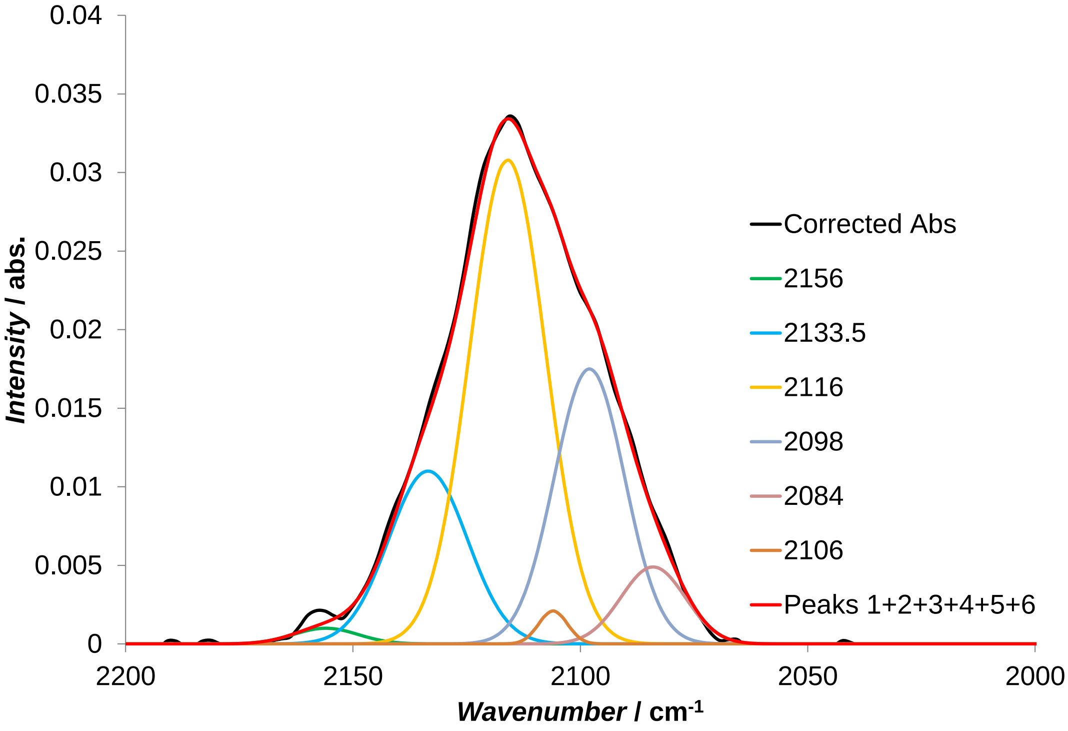

0.04
0.035
0.03
0.025
0.02
0.015
0.01
0.005
0
Intensity / abs.
2200
2150
2100
2050
2000
Wavenumber / cm-1
Corrected Abs
2156
2133.5
2116
2098
2084
2106
Peaks 1+2+3+4+5+6

(E)

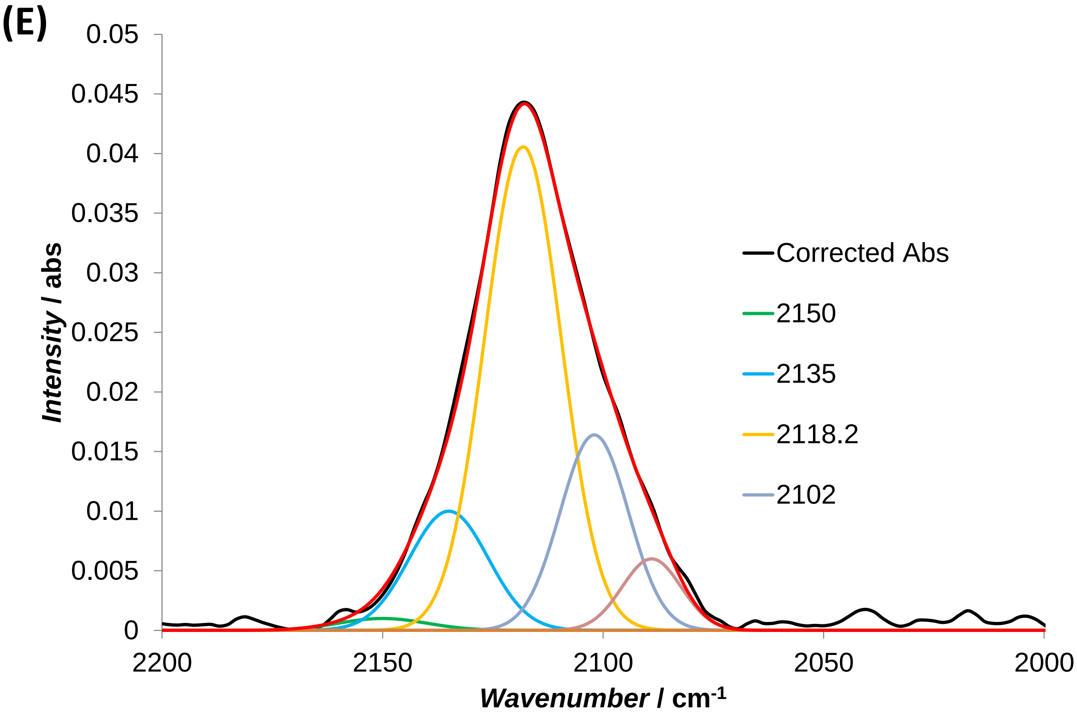

0.05
0.045
0.04
0.035
0.03
0.025
0.02
0.015
0.01
0.005
0
Intensity / abs
2200
2150
2100
2050
2000
Wavenumber / cm-1
Corrected Abs
2150
2135
2118.2
2102

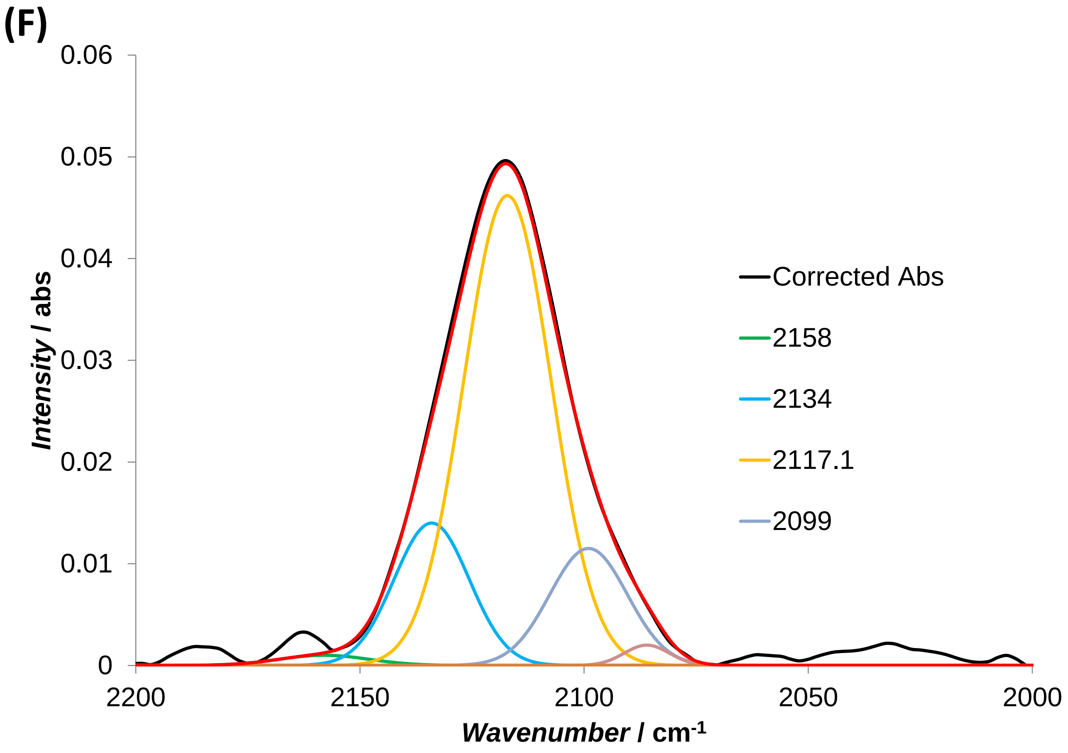

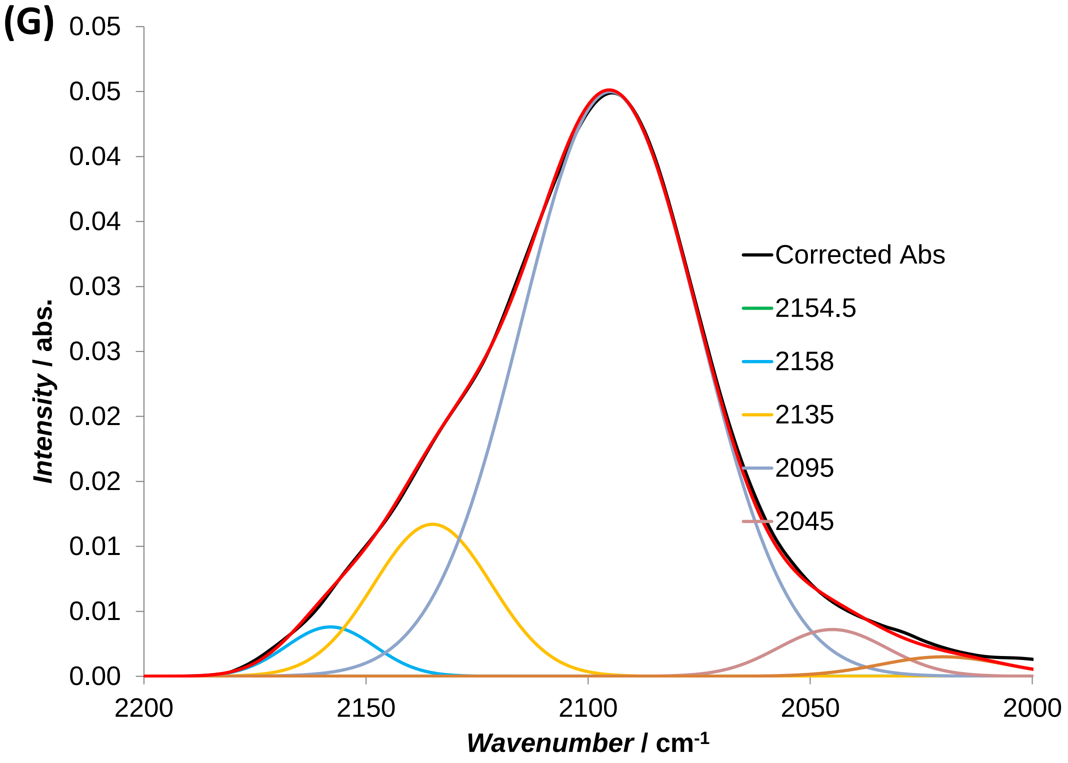

Figure S14: Gaussian peak deconvolution for plasma on (6 kV, 22.0 kHz) (A-C), plasma off (D-F) cycles and at 150 °C (G) in the simple WGS reaction mixture (2.0% CO, 7.5% H_2O) over 2% Au/$CeZrO_4$ catalyst. Gas phase CO subtracted.

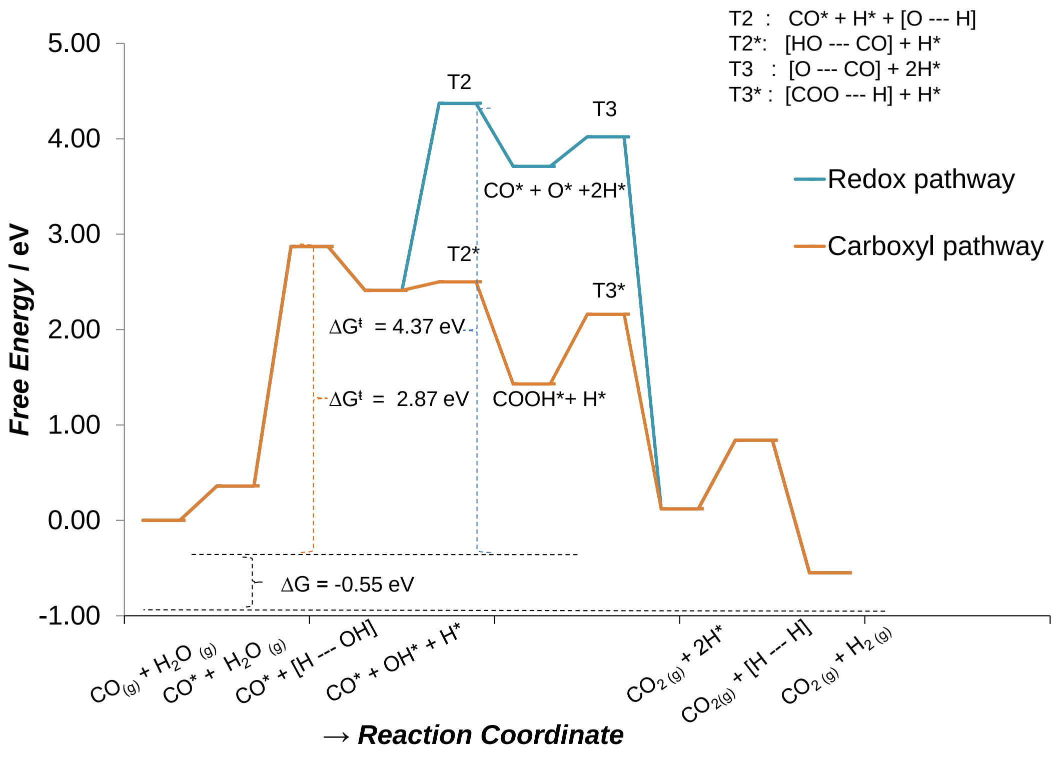

Figure S15: Free energy profile of WGS on Au(111) for both redox and carboxyl pathways.

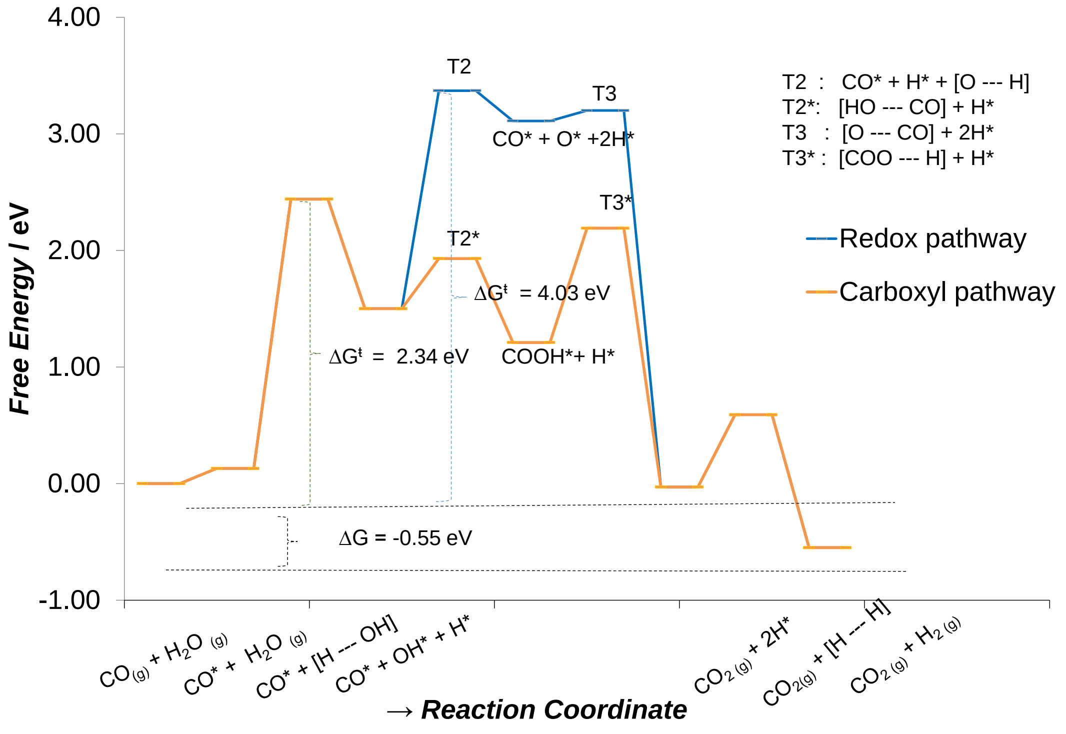

Figure S16: Free energy profile of WGS on Au(100) for both redox and carboxyl pathways.

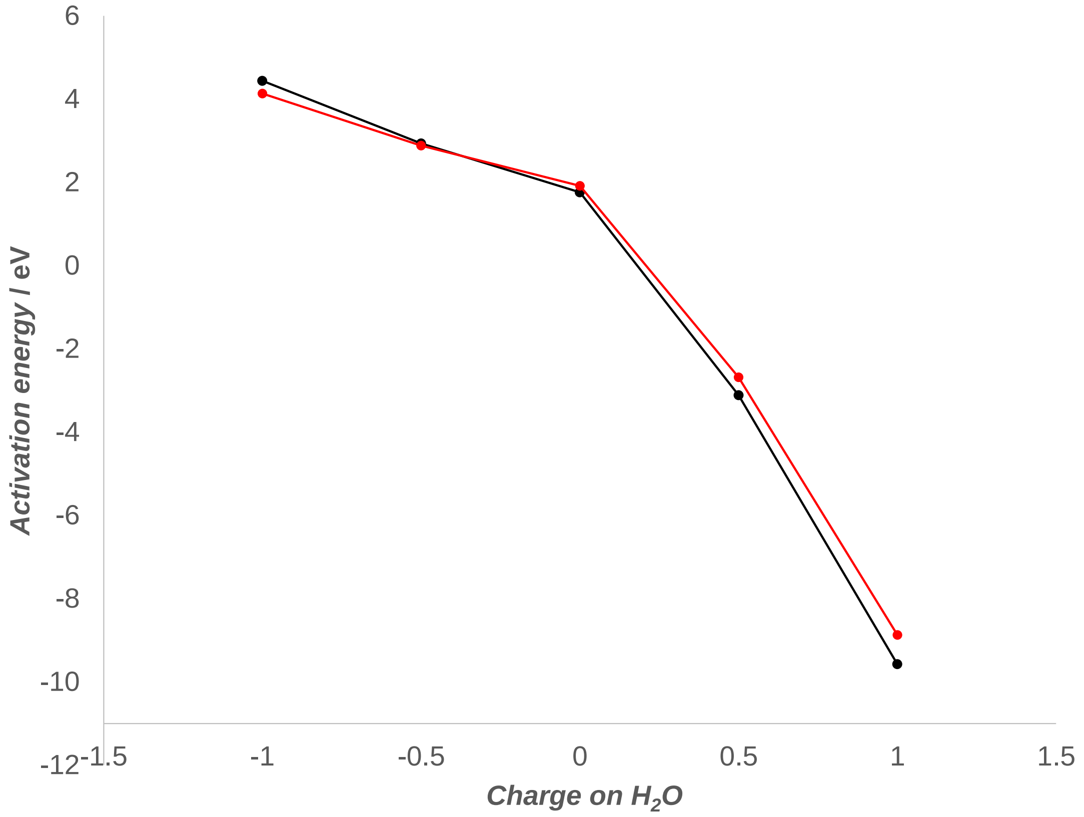

Figure S17: The relation between charges on gas-phase H_2O and the corresponding activation energies of H_2O dissociation on both Au(100) (black) and Au(111) (red).
